# HDAC4 in cancer: A multitasking platform to drive not only epigenetic modifications

**DOI:** 10.3389/fmolb.2023.1116660

**Published:** 2023-01-24

**Authors:** Emma Cuttini, Camilla Goi, Ester Pellarin, Riccardo Vida, Claudio Brancolini

**Affiliations:** ^1^ Scuola Superiore Universitaria di Toppo Wassermann, Università degli Studi di Udine, Udine, Italy; ^2^ Laboratory of Epigenomics, Department of Medicine, Università degli Studi di Udine, Udine, Italy

**Keywords:** HDACs, enhancers, H3K27ac, MEF2, sarcomas, senescence

## Abstract

Controlling access to genomic information and maintaining its stability are key aspects of cell life. Histone acetylation is a reversible epigenetic modification that allows access to DNA and the assembly of protein complexes that regulate mainly transcription but also other activities. Enzymes known as histone deacetylases (HDACs) are involved in the removal of the acetyl-group or in some cases of small hydrophobic moieties from histones but also from the non-histone substrate. The main achievement of HDACs on histones is to repress transcription and promote the formation of more compact chromatin. There are 18 different HDACs encoded in the human genome. Here we will discuss HDAC4, a member of the class IIa family, and its possible contribution to cancer development.

## 1 Introduction

The aim of this manuscript is to provide an updated overview of the recent progresses regarding the contribution of the epigenetic regulator HDAC4 to cancer development. However, to provide readers with a critical view of the sometime controversial evidence on HDAC4 and cancer, it is essential to discuss the complex networks of regulations, interactions, and signals that influence HDAC4 activities. Therefore, the first part of the review (Chapters 2-4) is devoted to learning about the basic mechanisms of HDAC4 regulation and its ability to interact with different partners. The second part (Chapter 5) is dedicated to HDAC4 in cancer, discussing the current state of research in hematological and solid tumors and considering the principal hallmarks of cancer (Hanahan et al., 2022).

### 1.1 The class IIa HDACs

Histone deacetylase 4 (HDAC4) belongs to the class IIa family of deacetylases, which includes HDAC4, HDAC5, HDAC7, and HDAC9. These epigenetic regulators contribute to the regulation of the lysine acetylation/deacetylation cycle by antagonizing the action of histone acetyl transferases (HAT/KAT). In vertebrates, class IIa HDACs have negligible enzymatic activity toward acetyl-lysine. Although they possess a deacetylase domain and bind the zinc ion required for catalysis, a substitution of the critical tyrosine residue by a histidine in the catalytic pocket was selected during evolution (Lahm et al., 2007). However, by assembling into multiprotein complexes, class IIa HDACs can act as a platform and coordinate the activity of class I HDACs (Brancolini et al., 2022). The reason for this evolutionary selection is unclear. Some hypotheses have been formulated, such as the role of the deacetylase domain as a reader of acetylated histones to localize class I HDACs in competent chromatic environments. Other hypotheses include the possibility of activity against yet unknown post-translational modifications (PTMs) of lysine, which in principle, should be bulkier because of the larger catalytic pocket. Certainly, further studies are needed to clarify this still enigmatic trait of class IIa HDACs. Studies that are also critical for the development of specific inhibitors of class IIa HDACs.

Like other epigenetic regulators, the activity of class IIa HDACs is subject to tight control of various extracellular signals that allow cells to adapt their activities to the needs of the organism. Consequently, the activity of class IIa HDACs is regulated at multiple levels, including transcription, translation, and various PTMs, with phosphorylation playing an important role. Phosphorylation of class IIa controls mainly protein stability and the nuclear-cytoplasmic shuttling. Frequently, these events are responsible for the removal of the repressive influence of HDACs on gene transcription (Wang, and Yang, 2001; Cernotta et al., 2011; Wang Z et al., 2014). Because of these multiple levels of regulation, it is not easy to identify the specific genetic alterations that may be responsible for affecting HDAC4 activities during tumorigenesis. Before discussing possible contributions of HDAC4 to cancer development and aggressiveness, we will review some basic concepts about HDAC4.

## 2 The *HDAC4* gene

**FIGURE 1:**
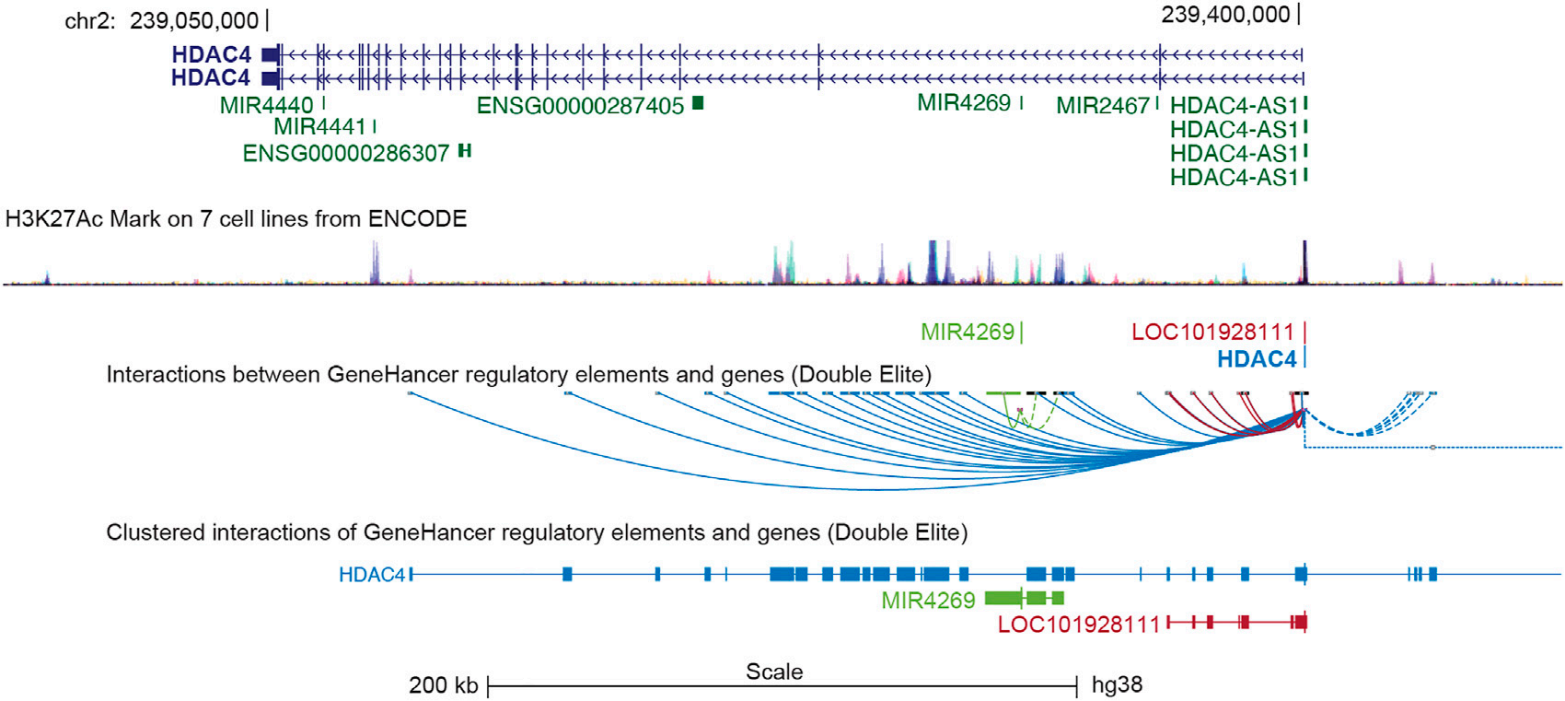
The genomic organization of the HDAC4 locus. The position of the different transcripts is indicated. HDAC4 organization in introns and exones is highlighted. The H3K27ac marks obtained from 7 cell lines of the encoded project are indicated to underline the regulative regions. LOC101928111 corresponds to HDAC4-AS1. Data were retrieved from https://genome.ucsc.edu/ UCSC Genome Browser on Human (GRCh38/hg38). The GeneHancer tool was selected to visualize putative enhancers and DNA loops.

In humans, the *HDAC4* gene is located on the minus strand of chromosome 2q37.3 and is approximately 350 kbp in length (Table 1 **)**. The gene is organized into 27 exons and 26 introns. Several, mostly unverified, transcript variants of HDAC4 (>40) with different lengths have been mapped. The isoforms of HDAC4 vary in length and include between 1016 and 1113 amino acid residues. Isoform 1, encoded by transcript variant 1 (NM _001378414.1 and NP _001365343.1), is 8,461 nucleotides (nt) long with a coding DNA sequence (CDS) of 3,270 nt and a corresponding protein length of 1,089 amino acids (aa). Various lncRNAs and miRNAs are embedded within the HDAC4 locus (Figure 1; Table 1). The HDAC4 locus is also characterized by the presence of regulatory elements, particularly enhancers, which can exert their influence both locally and through chromosomal loops at distant sites (Figure 1).

**TABLE 1:** The HDAC4 locus. Genes transcribed from the HDAC4 locus are indicated. Data were obtained from https://www.ncbi.nlm.nih.gov/gene/9759 (Ensembl release 107).

Accession	Start	Stop	Length (nt)	Gene symbol	Strand	Type	Name
NC_000002.12	239048168	239401654	353486	ENSG00000068024	Minus	mRNA	HDAC4
NC_000002.12	239068817	239068914	97	ENSG00000266109	Minus	miRNA	MIR4440
NC_000002.13	239085827	239085926	99	ENSG00000264810	Minus	miRNA	MIR4441
NC_000002.14	239114858	239118842	3984	ENSG00000286307	Plus	LncRNA	
NC_000002.15	239194118	239197654	3536	ENSG00000287405	Plus	LncRNA	
NC_000002.16	239305462	239305545	83	ENSG00000265215	Plus	miRNA	MIR4269
NC_000002.17	239351724	239351804	80	ENSG00000264292	Minus	miRNA	MIR2467
NC_000002.18	239401436	239402657	1221	ENSG00000222020	Plus	LncRNA	HDAC4-AS1
NC_000002.19	239439595	239531363	91768	,ENSG00000286525	Plus	LncRNA	

In several tissues, HDAC4 is the lowest expressed class IIa HDAC after HDAC9. Exceptions are bladder, colon, esophagus, and uterus. The ENCODE project has revealed that several transcription factors (TFs), epigenetic modifiers and architectural proteins bind to the proximal promoter of HDAC4, suggesting that HDAC4 transcription is indeed heavily regulated (Rosenbloom et al., 2009; Di Giorgio and Brancolini, 2016). Among these TFs some proto-oncogenes can be found, such as JUN, FOS, and MYC, which control the G0/G1 transition. For further information, the reader is kindly invited to consult (Di Giorgio and Brancolini 2016).

Within a large deletion on chromosome 2q37, haploinsufficiency of the HDAC4 gene has been reported to cause the 2q37 deletion syndrome, a disorder with significant intellectual impairment, brachydactyly type E (BDE), and typical facial features (Williams et al., 2010). Further studies have shown that haploinsufficiency of HDAC4 for BDE is not completely penetrant (Villavicencio-Lorini et al., 2013) and is not sufficient to cause intellectual disability (Wheeler et al., 2014). More recently, heterozygous *de novo* missense variants affecting amino acid residues involved in phosphorylation-dependent binding of 14-3-3 proteins (see below) and control of the nucleocytoplasmic shuttle, have been identified in individuals with delayed developmental milestones, intellectual disability and hypotonia, a phenotype distinct from 2q37 deletion syndrome (Wakeling et al., 2021). Although HDAC5 is the predominant family member in the central nervous system (Brancolini et al., 2021), the role of HDAC4 in controlling synaptic gene expression and the alterations in neurotransmission, learning, and memory observed in some but not all mouse models with dysregulated HDAC4 support a possible role in central nervous system (CNS) functions (Wu et al., 2016).

Genetic studies in mice have demonstrated the contribution of *Hdac4* to various differentiation and adaptation responses. *Hdac4* plays an irreplaceable role in controlling chondrocyte hypertrophy and limiting premature ossification of endochondral bone (Vega et al., 2004). A phenotype that depends in part on the upregulation of MMP13 (Nakatani et al., 2016). Tissue-specific *Hdac4* knockouts (KOs) have demonstrated a key role of the deacetylase in controlling satellite cell proliferation and mediating the skeletal muscle response to denervation (Choi et al., 2014; Marroncelli et al., 2018; Pigna et al., 2018). In the liver, deletion of *Hdac4* alters the regulation of glycogen storage (Mihaylova et al., 2011). Since Hdac4 is a member of the class IIa HDAC family, its role in other biological responses may be underestimated due to redundancy, especially with the phylogenetically closest member, HDAC5. In addition, genetic compensatory mechanisms monitored by Jun and Mef2 may increase the expression of one family member when deficiencies occur in other members (Velasco-Aviles et al., 2022). Recruitment of HDAC4 at a specific genomic locus can trigger a fast gene silencing (Lensch et al., 2022). However, certain loci seem to be refractory to HDAC4 repressive activity even though artificially deposited through the Cas9 delivery system (Di Giorgio et al., 2021). A result that points to the local chromatin environment as a licensing factor for HDAC4 activity.

## 3 The HDAC4 protein

**FIGURE 2:**
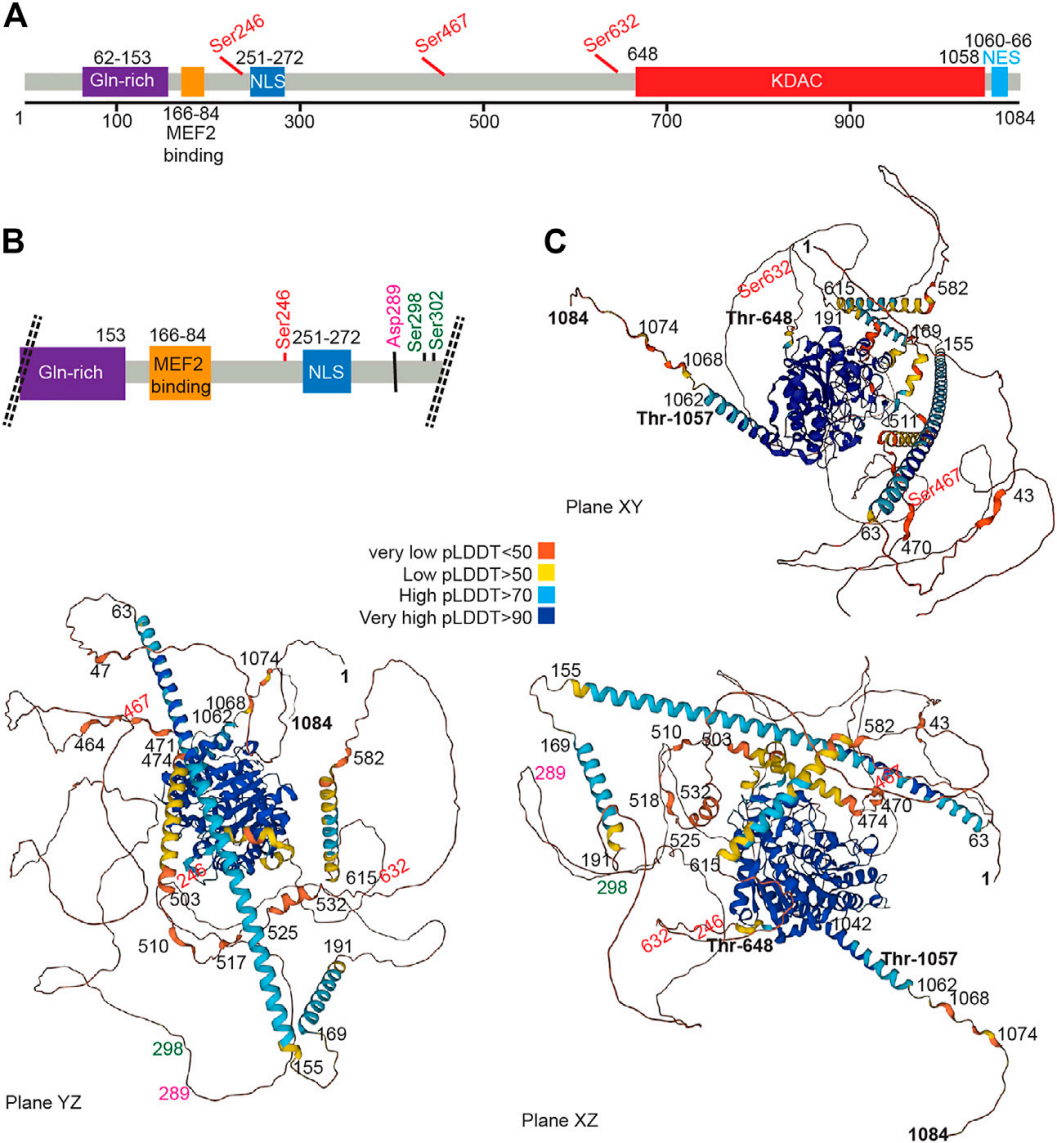
The HDAC4 protein. **(A)** Schematic view of the HDAC4 protein. The major domain and regulative aa sites are highlighted. **(B)** Schematic view at higher magnification of the N-terminal region of HDAC4. **(C)** AlphaFold prediction of HDAC4 structure. Three different views are provided. Colors indicate the different per-residue confidence score (pLDDT) as indicated. Some regions below 50 pLDDT may be unstructured in isolation. https://alphafold.ebi.ac.uk/entry/P56524.

The HDAC4 protein (Figure 2) consists of a long N-terminal region responsible for protein interactions and a highly conserved C-terminal lysine deacetylase (KDAC) domain. The N-terminal region contains a lysine/arginine-rich nuclear localization sequence (NLS) spanning residues 244–279 and binding sites for transcription factors or other co-repressors (Di Giorgio et al., 2015), including members of the MEF2 family (myocyte enhancer factor-2), which was mapped to residues 166–184, (Miska et al., 1999; Wang et al., 2000). According to structural studies, this sequence can be folded into an *α*-helix that fits into a hydrophobic groove on the surface of a MEF2 dimer (Minisini et al., 2022). Like several other epigenetic regulators, HDAC4 cannot bind to DNA in a sequence-dependent manner (Wang et al., 2013; Torchy, et al., 2015; Hosokawa and Rothenberg, 2021). Interaction with TFs provides a strategy to recruit deacetylase to a specific region of the genome and alter chromatin. Alternative strategies can also be pursued. HDAC4, by joining multiprotein complexes containing epigenetic readers, can localize close to nucleosomes with specific histone modifications. Finally, HDAC4 and class IIa HDACs may themselves function as readers because of their low deacetylase activity, although they have not been studied in detail.

The N terminus of HDAC4 also associates with chaperone proteins, including the 14-3-3 protein, and this association allows nuclear export, thereby abolishing the repression of HDAC target genes (Grozinger and Schreiber, 2000; Wang et al., 2000; Wang B et al., 2014).

Early work has shown that overexpression of HDAC4 leads to aggregation in the nucleus and cytoplasm, suggesting the existence of a self-interaction domain (Miska et al., 1999). This hypothesis is confirmed by the presence of two domains with a high probability of forming coiled-coil structures, encompassing amino acids 67–150 and 173–184, as shown by the AlfaFold software (Jumper et al., 2021; Varadi et al., 2022). The N-terminal domain contains a glutamine-rich domain that can fold into a straight alpha helix that assembles into a tetramer (Guo et al., 2007). Accordingly, N-terminal deletion mutants show an inability to self-bind, supporting the notion that the N-terminal domain of HDAC4 contains an oligomerization domain (Kirsh et al., 2002).

The carboxy-terminal region contains the deacetylase domain with a hydrophobic pocket in which the zinc ion is coordinated. The zinc-containing domain of HDAC4 consists of residues from T648 to T1057 (Bottomley et al., 2008). This domain interacts with the NCOR1/NCOR2/HDAC3 complex, which can provide the deacetylase activity (Guenther et al., 2001; Di Giorgio et al., 2015; Hudson et al., 2015). A repeated peptide motif presents in both NCOR1 and NCOR2 is sufficient to mediate interaction with HDAC4. This peptide sequence binds near the active site of HDAC4 and requires the “closed” conformation of the zinc-binding loop on the surface of the enzyme (Hudson et al., 2015).

In addition, a hydrophobic nuclear export sequence (NES) is located between residues 1051 and 1084 at the C-terminal end of HDAC4 (Wang et al., 2000; McKinsey et al., 2001; Mathias et al., 2015). This sequence is required for CRM1-dependent nuclear export of HDAC4 and its accumulation in the cytoplasm.

### 3.1 Control of nucleocytoplasmic shuttling: (de)phosphorylation

HDAC4 is subject to microenvironment-dependent regulation through the action of several PTMs, which include phosphorylation, SUMOylation, and proteolytic cleavages (Mathias et al., 2015). Most of the documented PTMs control nuclear-cytoplasmic transport, the major process regulating the corepressor functions of HDAC4 (Table 2). Control of nuclear-cytoplasmic shuttling is a common strategy to influence the activities of class IIa HDACs and offers advantages in terms of response time and adaptability (Chen et al., 2020).

**TABLE 2:** Main PTMs regulating HDAC4 activities.

PTM	Enzyme		Site	Effect	Reference: DOI
Phosphorylation	CamK family	CamKI	S246, S467	Nuclear export	Backs et al. (2006)
		CamKII	S467, S632	Nuclear export	Backs et al. (2006)
		CamKIV	S467, S632	Nuclear export	Zhao et al. (2001)
		CamKdB	S210	MEF2 silencing	Little et al. (2007)
	PKD1		S246, S467	Nuclear export	Sinnett-Smith et al. (2014)
	EMK		S246	Cytoplasmic retention	Dequiedt-Smith et al. (2006)
	CTAK1		S246	Cytoplasmic retention	Dequiedt-Smith et al. (2006)
	AMPK		N/A	Nuclear export	Jiang et al. (2020)
	SIKs	SIK1, SIK2, SIK3	S246, S467, S632	Nuclear export	Berdeaux et al. (2007), Walkinshaw et al. (2013)
	FAK		N/A	N/A	Sato et al. (2020)
	GSK3b		S298, S302	UPS-mediated degradation	Cernotta et al. (2011)
	PKA		S265, S266, S584	Nuclear retention	Liu and Schneider (2013), Doddi et al. (2019)
	Aurora B kinase		S265	N/A	Guise et al. (2012)
	PP2A		S246, S467, S632, S298	Nuclear import	Paroni et al. (2008)
SUMOylation	RanBP2		K559	Nuclear retention	Kirsh et al. (2002)
Proteolitic cleavage	Caspase-2 Caspase-3		D298	Apoptosis	Liu et al. (2004), Paroni et al. (2004)
	PKA-dependent		Between Y201 and W202	Inhibition of MEF2	Backs et al. (2011)
Ubiquitylation	N/A		N/A	UPS-mediated degradation	Cernotta et al. (2011), Potthoff et al. (2007)

Phosphorylation at S246, S467, and S632 creates docking sites for 14-3-3 chaperone proteins that promote the translocation of HDAC4 from the nucleus to the cytoplasm. Because HDAC4 localization is associated with transcriptional regulation, the loss of these phosphorylation sites enhances the transcriptional activity of MEF2 (Di Giorgio et al., 2015; Mathias et al., 2015; Chen et al., 2020). Cytoplasmic sequestration appears to be caused by NLS masking because of 14-3-3 protein binding. Binding that prevents association with the importin α/β heterodimer is an obligatory step for HDAC4 nuclear import (Grozinger and Schreiber, 2000).

Many isoforms of the calcium/calmodulin-dependent kinase (CaMK) family phosphorylate HDAC4 and inhibit its accumulation in the nucleus. CaMKI preferentially phosphorylates S246. CaMKII phosphorylates residues S467 and S632 on HDAC4 by binding to a unique docking site (centred on Arg 601) that is not present in other class IIa HDACs (Backs et al., 2006; Parra and Verdin, 2010). The same serines are also phosphorylated by CaMKIV, which can also promote nuclear export by a mechanism independent of 14-3-3 protein binding (Zhao et al., 2001; Yuan et al., 2016). In cardiac cells, CaMKIIδB was described to preferentially target residue S210 of HDAC4 rather than the other HDACs of the class IIa. It was hypothesized that this phosphorylation might cause a conformational change for the recruitment of additional factors that mediate MEF2 silencing. (Little et al., 2007).

Protein kinase A (PKA) phosphorylates S265 and S266 in cardiac and skeletal muscle cells, resulting in decreased HDAC4 efflux from the nucleus (Liu and Schneider, 2013). PKA can also phosphorylate serine 584. In this case, the effects are less clear, although an increase in the repressive activity of HDAC4 toward MEF2 is plausible (Doddi et al., 2019). Interestingly, parathyroid hormone (PTH) induces PKA-dependent phosphorylation of S740, leading to the export of HDAC4 to the cytoplasm and its degradation *via* a lysosomal-dependent system (Shimizu et al., 2014). In the heart, coordinated actions of PKA and CaMKII regulate the entry and exit of HDAC4 into the nucleus (Helmstadter et al., 2021).

Importantly, PKA in macrophages could also indirectly affect the repressive activity of HDAC4 by inhibiting salt-inducible kinases (SIKs) (Luan et al., 2014).

Other kinases are involved in the phosphorylation of HDAC4, pointing to HDAC4 as an hub for different signalling pathways. An exhaustive list is provided in Table 2. Protein kinase D1 (PKD1) phosphorylates residues S246 and S467, promoting nuclear export and cytoplasmic retention of HDAC4 (Sinnett-Smith et al., 2014; Pablo Tortola et al., 2021).

MARK/Par-1 kinases such as EMK and C-TAK1 have been described to control HDAC4 localization by phosphorylating S246. This facilitates phosphorylation of the remaining residues required for 14-3-3 binding (S467 and S632) by other kinases, resulting in cytoplasmic retention (Dequiedt et al., 2006).

AMP-activated protein kinase (AMPK), the major sensor of energy metabolism, can phosphorylate HDAC4 and HDAC5, to promote their nuclear exclusion and the epigenomic resetting. (Salminen et al., 2016; Niu et al., 2017).

In addition, salt-inducible kinase (SIK) subfamily activity is also involved in HDAC4 re-localization (Walkinshaw et al., 2013). During food intake, HDAC4 is phosphorylated and sequestered in the cytoplasm by SIK3, whose activity is upregulated in response to insulin, whereas the kinase is inactivated during fasting, leading to dephosphorylation and nuclear translocation of HDAC4. SIK2 mediates the phosphorylation and inactivation of HDAC4 in mouse hepatocytes in response to insulin. Conversely, glucagon exposure increases HDAC4 activity through PKA-mediated inhibition of SIK2 (Wang et al., 2011). Both SIK2 and SIK3 phosphorylate HDACs at the conserved motifs for 14-3-3 binding and stimulate their nuclear export, thereby supporting MEF2-dependent transcription. However, unlike SIK2, SIK3 induces nuclear export independently of kinase activity and 14-3-3 binding (Walkinshaw et al., 2013). In muscle cells, SIK1 phosphorylates class IIa HDACs and promotes their export from the nucleus to the cytoplasm (Berdeaux et al., 2007).

Focal adhesion kinase (FAK)-mediated tyrosine phosphorylation controls the subcellular localization of HDAC4/5. Residue Y642 has been identified as the FAK-dependent tyrosine phosphorylation site for HDAC5, the closest member of the deacetylase family to HDAC4, but the precise role of Y642 phosphorylation of HDAC5 in controlling its subcellular localization remains to be determined (Sato et al., 2020).

Glycogen synthase kinase 3β (GSK3β) phosphorylates S298 and S302 and plays an important role in controlling the stability of HDAC4 (Cernotta et al., 2011; Wang Z et al., 2014; Xiao Q et al., 2021). S302 may serve as a priming phosphorylation site that promotes the subsequent phosphorylation of HDAC4 at S298. This phosphorylation provides a signal for poly-ubiquitylation; therefore, phosphorylation of HDAC4 by GSK3β promotes UPS-mediated degradation of HDAC4 during growth arrest and senescence (Cernotta et al., 2011; Di Giorgio et al., 2021).

Control of HDAC4 and other class IIa HDACs during mitosis can also be exploited by phosphorylation. In this case, it is the Aurora B kinase that phosphorylates serine 265 within the NLS. This results in decreased association with HDAC3 and impaired repression of transcription. However, it is unclear whether other functions, unrelated to transcriptional regulation, may be affected (Guise et al., 2012). Interestingly, the role of HDAC4 in chromosome segregation has been described in TP53-defective cells (Cadot et al., 2009), which may be related to Aurora B-dependent phosphorylation.

Phosphorylation of HDAC4 is reversible, and the removal of phosphate groups is mediated by the protein phosphatase 2 A (PP2A) family, which promotes the accumulation of HDAC4 in the nucleus. Specifically, the N-terminus of HDAC4 interacts with the catalytic subunit of PP2A, which dephosphorylates several serines, including 14-3-3 binding sites and S298, enabling nuclear import of HDAC4 (Paroni et al., 2008; Veloso et al., 2019; Tan et al., 2022).

Finally, a combinatorial mass spectrometry approach revealed that HDAC5 has at least 17 *in vivo* phosphorylation sites within functional domains, including NLS, NES, and KDAC domains. These novel phosphorylation sites suggest the existence of additional unexplored phosphorylation-dependent mechanisms that dynamically regulate class IIa HDACs (Greco et al., 2010). Considering that HDAC5 is the closest member of the deacetylase family to HDAC4, mass spectrometry may provide comparable results for HDAC4, suggesting novel multiple regulatory mechanisms of the deacetylase.

### 3.2 HDAC4 protein stability

HDAC4 protein stability is regulated by the ubiquitin-proteasome system. HDAC4 polyubiquitylation is a widely used mechanism for the radical silencing of all HDAC4 activities (both cytoplasmic and nuclear) (Renzini et al., 2022). HDAC4 polyubiquitylation has been observed under various conditions: In response to growth factor deprivation, during the onset of senescence, during hypoxia, in response to alcohol consumption in the brain or viral infection (Potthoff et al., 2007; Cernotta et al., 2011; Du et al., 2015; Griffin et al., 2017; Lu et al., 2019; Di Giorgio et al., 2021).

In osteoblasts HDAC4 serves as a brake for differentiation into osteoclasts. HDAC4 degradation is triggered by parathyroid hormone (PTH) to allow MEF2C-dependent transcription and RANKL expression. In this case, it has been proposed that the E3-ligase SMURF2 plays a role (Obri et al., 2014).

Other studies have suggested that HDAC4 levels may be under the control of lysosomal proteases. PTH leads to the export of HDAC4 to the cytoplasm through PKA-dependent phosphorylation of S740 and its degradation *via* a lysosomal-dependent system (Shimizu et al., 2014). Under excessive oxidative stress, a lysosomal serine protease released from disrupted lysosomes can generate an N-terminal fragment of HDAC4. This fragment triggers chaperone-mediated autophagy degradation of MEF2A and neuronal cell death (Zhang et al., 2014).

The involvement of lysosomes in the regulation of HDAC4 challenges the autophagic response. HDAC4 has been reported to both suppress and stimulate autophagy (Kang et al., 2014; Yue et al., 2015; Pigna et al., 2018; Yang et al., 2018). The involvement of HDAC4 in regulating autophagy may also be part of feedforward circuits that maintain malignancy (Zang et al., 2022). The relationships between HDAC4 and autophagy are complex and require further investigation. It needs to be clarified whether the influence of HDAC4 on autophagy is direct or the result of cellular stresses induced by experimental manipulation of HDAC4 levels.

Artificially manipulating HDAC4 levels may represent an alternative strategy to the use of inhibitors from a clinical perspective. Recently, the first bifunctional protein degraders of HDAC4 have been developed using proteolysis targeting chimeras (PROTACs) technology (Macabuag et al., 2022). AUTOphagy-TArgeting Chimeras (AUTOTACs) to degrade HDAC4 *via* the macroautophagy pathway may also represent a promising strategy (Ding et al., 2022).

### 3.3 Additional modifications

Although phosphorylation is the main PTM of HDAC4 studied, other additional modifications may also influence HDAC4 activity and localization.

Reactive oxygen species (ROS) may control the subcellular localization and activity of HDAC4, although the mechanisms involved are not completely clear (Wang B et al., 2014; Di Giorgio and Brancolini, 2016; Schader et al., 2020).

Selective proteolysis can modulate the activity of HDAC4. Caspase 2 and caspase 3 cleave HDAC4 at D289 and generate an NLS-containing fragment with selective repressive activities (Paroni et al., 2007; Cao K et al., 2014; Zhou J et al., 2015). Phosphorylation by PKA may also render HDAC4 competent for cleavage by the serine protease under the influence of ABHD5. This N-terminal fragment represses MEF2-dependent transcription and protects mice from heart failure (Jebessa et al., 2019).

HDAC4 can be SUMOylated to lysine 559. A modification that likely occurs during translocation through the nuclear pore and enhances its repressive influence (Kirsh et al., 2002). It has been suggested that HDAC4 may regulate SUMOylation of other proteins such as IkBα, DACH1, and LXR proteins as part of a yet not clearly defined molecular complex (Lee et al., 2009; Ozcan et al., 2016; Yang et al., 2020), HDAC4 interacts with the E2 ligase Ubc9 and appears to be responsible for E3 ligase activity. An activity that may also play a role during senescence or in DNA repair (Dehennaut et al., 2013; Han et al., 2013).

## 4 HDAC4 as a platform to coordinate multiple tasks

**FIGURE 3:**
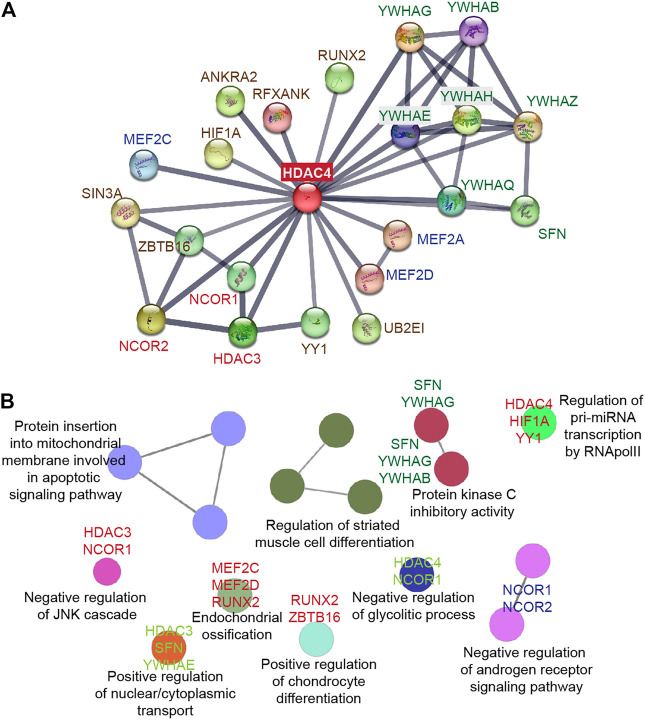
HDAC4 as a platform to orchestrate multiple protein interactions. **(A)** STRING protein-protein interaction (PPI) network (Szklarczyk et al., 2021) was created to capture HDAC4 molecular partners. **(B)** The biological functions of the HDAC4 partners were analyzed using the ClueGO plug-in from Cytoscape (Bindea et al., 2009).

The N-terminal domain of HDAC4 is a distinctive and crucial feature of class IIa deacetylases. It is responsible for homomeric interactions and is required for interactions with multiple partners, including transcription factors, co-repressors, and histone-modifying enzymes (Figure 3).

### 4.1 Interaction with corepressor complexes mediates histone deacetylation

In vertebrates, the HDAC4 protein is enzymatically inactive, so deacetylase activity on histone proteins depends on the formation of a protein complex containing HDAC3, a class I histone deacetylase bound to NCOR1 and NCOR2 to form the active heterotrimer. The deacetylase domain of HDAC4, particularly the edge of the hydrophobic channels leading to the catalytic site, is involved in this interaction (Kim et al., 2015). In NCOR1/NCOR2, the site of interaction is repression domain 3 (RD3), which interacts specifically with class IIa and not class I HDACs. The RD3 domain is predicted to be intrinsically disordered and has been assigned to a conserved and repeated motif of eight amino acids, the GSI (GSI(S/T)XGXP) motif. GSI requires that the specific class IIa loop adopt the so-called “closed” configuration. Importantly, HDAC inhibitors disfavor binding to the corepressor and instead favor the open configuration of the class IIa specific loop (Hudson GM et al., 2015; Park et al., 2018).

These observations strongly suggest that HDAC4 and class IIa HDACs have evolved into pseudoenzymes that have a structural rather than a catalytic function. However, they also suggest that inhibitors of class IIa HDACs may promote acetylation, by acting as allosteric inhibitors and interfering with the binding of HDAC4 to the NCOR1/NCOR2/HDAC3 complex, even though these deacetylases no longer have catalytic activity (Hudson GM et al., 2015; Park et al., 2018).

HDAC4 activity is essential for HDAC3-mediated deacetylation of selected targets (Mihaylova et al., 2011; Lee et al., 2015), suggesting that its role as a scaffold protein is necessary for recruitment of additional functional partners.

Finally, early studies have indicated that the common splice version of HDAC9, MITR, can recruit HDAC1 *via* the N-terminal region (Sparrow et al., 1999). Although molecular details are lacking, a similar interaction cannot be ruled out for other class IIa HDACs.

### 4.2 Interaction with transcription factors ensures DNA binding specificity

Like the other HDACs, HDAC4 does not have a DNA-binding domain, and its recruitment to specific genomic sites can be mediated by interaction with selected TFs. Of the TFs described to form complexes with HDAC4, little information are available about the bound genomic regions (Mihaylova et al., 2011; Wang Z et al., 2014; Chen and Sang 2016; Kim et al., 2016; Li et al., 2019). Thus, we do not have a complete idea of whether some TFs interacting with HDAC4, are simply substrates of the deacetylase complex, or act as Trojan horse to localize HDAC4 in specific genomic regions. In some cases, both conditions might even apply (Zhao et al., 2005).

The foremost investigated example regards the MEF2 TFs. MEF2 belongs to a family of TFs characterized by the presence of a MADS box (MCM1, Agamous, Deficiens, Serum Response Factor Box) involved in different developmental pathways. In vertebrates, the family includes four paralogous genes, MEF2A, MEF2B, MEF2C, and MEF2D, which are involved in the control of a variety of biological functions depending on the cell type, including apoptosis, cell survival, proliferation, hypertrophy (Taylor and Hughes, 2017; Di Giorgio et al., 2018; Assali et al., 2019). HDAC4 has affinity for MEF2 *in vitro* and *in vivo* thanks to the binding of its N-terminal domain to a highly conserved region of MEF2 family members located at the junction of the MADS -box and the MEF2-specific domain (Lu et al., 2000). HDAC4 represses MEF2 transactivation through several mechanisms, including: 1) recruitment of corepressor complexes NCOR1/NCOR2/HDAC3 (Guenther et al., 2001), 2) competition for binding with transactivators such as p300 (Clocchiatti et al., 2013), 3) phosphorylation-dependent sumoylation (Grégoire et al., 2006).

The transcription factor HIF1 is the master regulator of oxygen homeostasis and consists of the subunits HIF1A and HIF1B. Under oxygen-rich conditions, HIF1A is constantly proteasomally degraded by recruiting a substrate recognition element, Von Hippel-Lindau protein (VHL), which promotes the action of an E3 ubiquitin ligase. Hypoxic conditions selectively suppress this degradation because the absence of prolyl and asparaginyl hydroxylation in HIF1A at critical residues inhibits recognition by the VHL protein (Semenza, 2012; Semenza, 2016). An interaction between HIF1A and HDAC4 has been reported. Functionally, HDAC4 regulates the posttranslational processing of HIF1A through the HSP70/HSP90 system, although, the closest family member HDAC5, seems to be more involved in this task (Chen and Sang, 2016).

### 4.3 Integrated models for HDAC4-dependent chromatin modulations: The platform and the positional effects

HDACs play an intermediate role in the final decision of the chromatin state at specific loci. By removing the acetyl group, they reset the epigenetic status and the pattern of protein complexes that are bound to histones and contemplates the activation of transcription. Furthermore, deacetylation can favor the intervention of other enzymes that stabilize a repressive outcome, through a wave of repressive methylations. Alternatively, Lysine acetyl transferases (KATs) can dynamically re-establish an open chromatin conformation and gene transcription.

Generally, histone deacetylation is followed by the onset of repressing modifications (e.g., H3K9me2, H3K27me3) which promote the recruitment of additional repressors to further condensate the nucleosome structure. The final goal is to impede the binding of TFs and to prevent transcription (Aloia, 2022; Franklin et al., 2022).

Overall, it seems evident that the general mechanism of HDAC4-induced transcriptional repression involves the assembly of multiprotein complexes, where HDAC4 acts as a recruitment platform and directs the activity of TFs and/or of epigenetic regulators (Hohl et al., 2013.; Di Giorgio et al., 2017).

An interesting model that supports this hypothesis was proposed by Finke et al. (2022) thanks to the genetic deletion of HDAC4 in adult mouse ventricular myocytes. The loss of HDAC4 led to a whole epigenome activation, with an increase of activating histone modifications (H3K4me3, H3K9ac and H3K27ac) and a decrease of repressing modifications (H3K9me2 and H3K27me3). Also, the CHIP-seq analysis revealed an overrepresentation of MEF2 binding sites in the upregulated regions characterized by the H3K9ac and H3K4me modifications. These findings prompt a model where HDAC4 acts as a scaffold protein that interacts with H3K9ac histones to recruit and direct enzymatically active partners—histone methyltransferase and class I HDAC to the genomic location specified by transcription factors, such as MEF2 (Finke et al., 2022).

Confirmation in the human setting and integration with additional classes of epigenomic regulators are found in a comprehensive genome-wide study carried out in cellular models of leiomyosarcoma (Di Giorgio et al., 2020). The study emphasized the importance of distal region binding in the mechanism of class IIa deacetylases-mediated epigenomic regulation. First, it was demonstrated that HDAC4 controls a peculiar genetic program and possesses both shared and specific genomic binding sites compared with HDAC9, resulting respectively in the modulation of MEF2D-regulated genes and non-MEF2D-regulated genes. It was observed that the two deacetylases mainly bind intergenic regions distal from the transcription start site (TSS). Instead, MEF2D is more frequently found at promoters. Furthermore, it was noticed that, even though a region is characterized by the co-presence of MEF2D*/*HDAC4*/*HDAC9 complexes, in some regions only one member plays an active role in the epigenetic regulation. This “*dominant positional effect”* was observed in an intergenic region distal from the AHRGEF28 locus, which shows features of an enhancer and regulates the expression of ENC1 thanks to chromatin looping. In fact, although both HDAC4 and HDAC9 bind this region, only HDAC9 knocked-out cells show an increase in H3K27ac levels at the enhancer and promoter sites, resulting in ENC1 upregulation.

Although H3K27ac is an important epigenetic mark under the regulation of HDAC4 and class IIa HDACs, additional marks correlate with HDAC4 activity such as lysine 9 in histone 3 (H3K9ac) (Ko et al., 2013; Zhao X et al., 2022).

Other studies have not found an involvement of HDAC4 in the regulation of the global levels of H4K36ac (Liu et al., 2015; Zhu et al., 2015). However, local activities cannot be excluded as well as the compensatory action of other family members. Finally, a still poorly investigated molecular complex formed by HDAC3-HDAC4 and emerin has been proposed to regulate H4K5 acetylation at a specific locus of a novel gene with anti-aging activity (NM_026333). This regulation was observed in the heart and contributes to the regulation of the autophagic response (Osanai et al., 2018).

## 5 HDAC4 dysregulations in cancer

As frequently observed for other epigenetic regulators, the contribution of HDAC4 to cancer seems to be context-dependent and therefore not easy to categorize (Bodily et al., 2011; Sandhu et al., 2012; Yim et al., 2013). Consequently, the question of whether HDACs and HDAC4 can act as tumor suppressors or oncogenes is debated (Brancolini et al., 2022). For example, high level of HDAC4 mRNA is an unfavorable prognostic marker in ovarian cancer, but favorable in pancreatic cancer (https://www.proteinatlas.org/ENSG00000068024-HDAC4/pathology). The molecular mechanism used by HDAC4 to exert its role in cancer is not completely understood (Wang B et al., 2014; Brancolini et al., 2022). It likely includes the interaction with the corepressor complexes NCOR1/NCOR2/HDAC3, that impacts on the transcriptional landscape of cancer cells through epigenomic resetting. Although plausible, the epigenomic mechanism does not exclude additional tasks, which independently form chromatin and DNA accessibility, are dysregulated in cancer cells in a HDAC4-dependent manner.

### 5.1 The landscape of genetic alterations. Is this enough?

**FIGURE 4:**
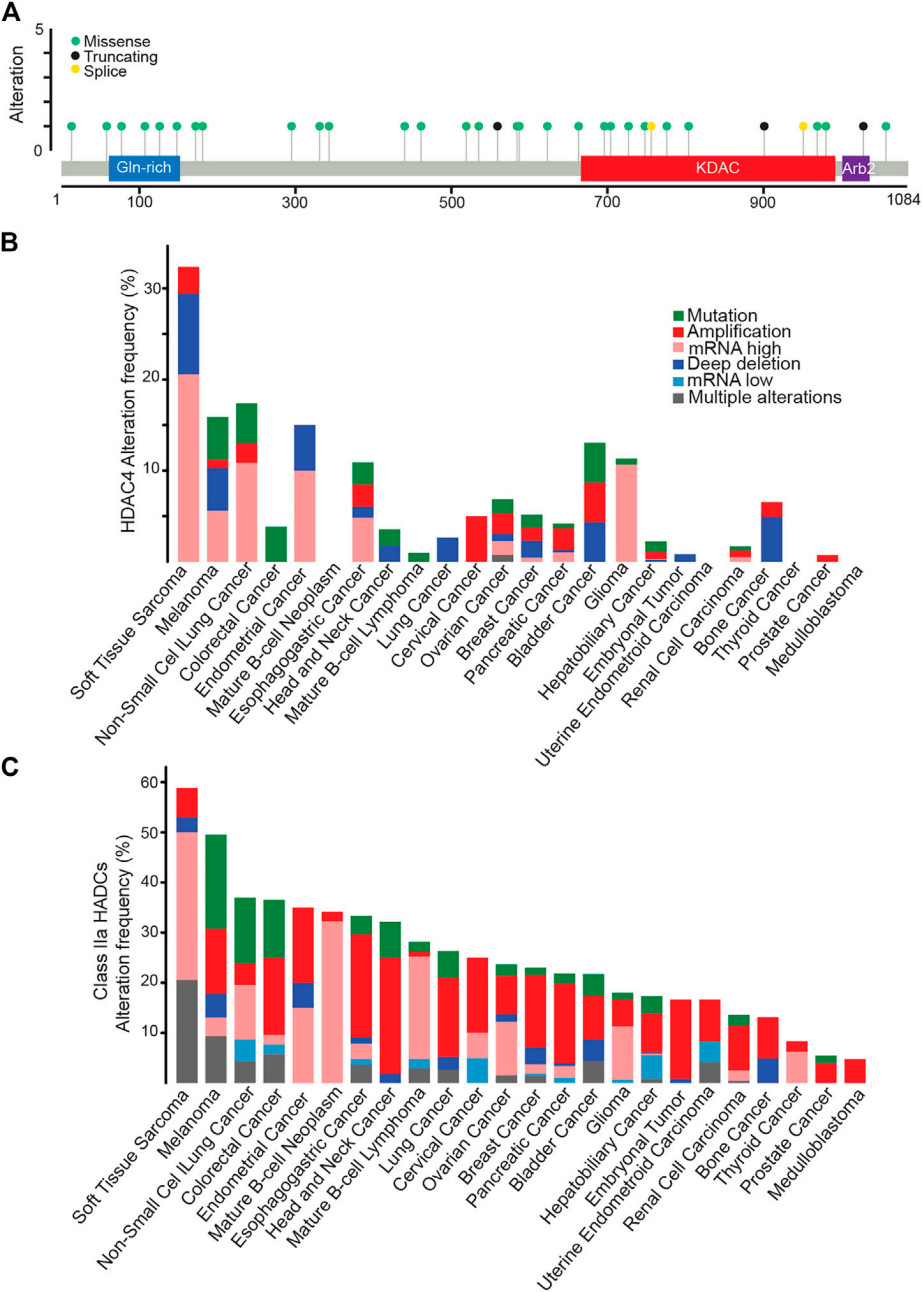
HDAC4 alterations in cancer. **(A)** Mutational profile of HDAC4. **(B)** Frequency of HDAC4 alteration in solid and liquid tumors. **(C)** Class IIa HDACs alteration frequency in solid and liquid tumors. Data were obtained from http://www.cbioportal.org. WGS changes cataloged in 2,583 whole-cancer genomes and their matched normal tissues across 38 tumor types. Source data from UCSC Xena and ICGC Data Portal.

Genetic alterations of HDAC4 in cancer mainly contemplate an increase in its expression level. Hot spot mutations are not evident and missense mutations are scattered throughout the protein. Curiously, the few splice variants and truncating mutations usually impact on the KDAC domain, leaving the amino-terminal part of the protein potentially expressed (Figure 4A). It is important to note that in the case of HDAC9 a splicing variant lacking the KDAC domain is highly expressed in different tissues (Brancolini et al., 2021). Therefore, it is plausible that truncated versions of HDAC4 might have a biological role in cancer.

The highest percentage of HDAC4 alterations (>30%) is found in soft tissue sarcoma (STS) (Figure 4B). A condition that holds true also when dysregulations of all members of the family are investigated (Figure 4C). Overall, almost 60% of STS patients present an alteration in a class IIa HDACs, as confirmed by previous observations (Di Giorgio et al., 2013; Di Giorgio et al., 2017; Di Giorgio et al., 2020). Point mutations are rare within this heterogenous group of tumors. Non-small cell lung cancer and melanoma are the second tumor types for frequency of genetic alterations in HDAC4 (15–17% of cases). Not surprisingly, these tumors are characterized by high mutational burden due to high exposition to carcinogens such as UV light or cigarette smoke (Klempner et al., 2020; Kalaora et al., 2022). Similarly, mutations in the mismatch repair pathway leading to microsatellite instability can explain the selective accumulation of HDAC4 point mutations in colorectal cancer patients (Klempner et al., 2020).

The dysregulation of HDAC4 in cancer could also involve the control of its subcellular localization as the result of alterations in cell signaling. Despite the importance of nuclear accumulation of HDAC4 in tumors, little data is available. Most cancers show moderate to strong cytoplasmic HDAC4 positivity (https://www.proteinatlas.org/ENSG00000068024-HDAC4/pathology) and some evidence support a correlation between the nuclear accumulation of HDAC4 and tumor aggressiveness (Di Giorgio et al., 2013). In several cultured cancer cell lines, HDAC4 shows a prominent cytoplasmic localization being actively exported from the nuclei. We also must consider that HDAC4 is subject to ubiquitin-proteasome system (UPS) mediated degradation and this event is maximized in the nuclear compartment (Cernotta et al., 2011). Whether a persistent nuclear localization of HDAC4 and the possible unrestrained repressive influence is incompatible with cell fitness is a fascinating hypothesis. Too less but also too much nuclear HDAC4 might be a deleterious condition.

### 5.2 Mechanisms of action: Is the control of proliferation the sole mechanism?

HDAC4 is frequently amplificated or overexpressed in cancer specimens and cell lines. As discussed above, *HDAC4* transcription is controlled by several oncogenes, including Jun, Fos, and Myc, arising the question of whether HDAC4 might be an oncogene itself (Di Giorgio and Brancolini, 2016). HDAC4 oncogenic activities have been proved in murine and human fibroblasts by classical *in vitro* transformation and oncogenic cooperation assays (Di Giorgio et al., 2013; Peruzzo et al., 2016; Paluvai et al., 2018). In these assays a mutant in the 14-3-3 binding sites that is predominantly localized in the nuclei shows a much stronger oncogenic activity. Supporting the putative oncogenic activity of HDAC4, mouse models with deregulated class IIa HDACs-HDAC7 or HDAC9-have been reported to develop cancer (Rad et al., 2010; Gil et al., 2016). The HDAC4 oncogenic pathway converges on the repression of MEF2 TFs. An action shared with PI3K signaling, which represses MEF2 activity through an independent route (Di Giorgio E et al., 2013). The ability of HDAC4 to repress the expression of tumor suppressor genes, such as CDKN1A, further supports a positive role of the deacetylase in cancer cells growth (Liu et al., 2009; Mottet et al., 2009; Clocchiatti et al., 2015). The fact that HDAC4 might be required to regulate cell proliferation is suggested by the result of high-throughput screenings using CRISPR/Cas9 technology (Table 3). In several CRISPR-based screenings performed in different cancer cell lines, HDAC4 emerges frequently as a significative hit required for cell fitness. It is possible that specific oncogenic transformations make cancer cells addicted to HDAC4 (Zhou et al., 2021). The dependency of some cancer cells from HDAC4 activity could be explained by its recently demonstrated role in the control of replicative senescence and oncogene-induced senescence (OIS) (see below, Di Giorgio et al., 2021).

**TABLE 3:** HDAC4 is required for the proliferation and survival of different cancer cells. Data of CRISPR screens and relative information were retrieved from the BioGRID ORCS open repository https://orcs.thebiogrid.org.

Cell type	Cell line	Method	Enzyme	Rank	Phenotype	PMID	Screen name
Acute myeloid leukemia	OCI-AML3	Knock-out	Cas9	1165/18663	Cell proliferation essential gene	28162770	9-PMID28162770
Acute myeloid leukemia	MOLM-13	Knock-out	Cas9	605/17995	Resistance to venetoclax	31048320	1-PMID31048320
Breast cancer	HCC1419	Knock-out	Cas9	1402/17670	Cell proliferation essential gene	29083409	72-PMID29083409
Cervical cancer	HeLa	Knock-out	Cas9	426/19113	Reduced proliferation in response to Olaparib	33257658	1-PMID33257658
Chronic myeloid leukemia	K-562	Knock-out	Cas9	89/14276	Extracellular vescicle production	30556811	2-PMID30556811
Colon cancer	HT55	Knock-out	Cas9	1679/17670	Cell proliferation essential gene	29083409	88-PMID29083409
Colon cancer	SW-620	Knock-out	Cas9	382/20111	Response to NK killer activity	34253920	3-PMID34253920
Gastric cancer	NCI-N87	Knock-out	Cas9	1120/17670	Cell proliferation essential gene	29083409	217-PMID29083409
Glioblastoma	U-138MG	Upregulation	dCas9-VP16	2/308	Resistance to temozomolide	33271924	2-PMID33271924
HIV latency	J-Lat A2	Knock-out	Cas9	389/446	Silencing latent retrovirus	31165872	1-PMID31165872
Lung cancer	A549	Knock-out	Cas9	1708/17670	Cell proliferation essential gene	29083409	6-PMID29083409
Lung cancer	NCI-H2172	Knock-out	Cas9	1577/17670	Cell proliferation essential gene	29083409	215-PMID29083409
Lung cancer	NCI-H1993	Knock-out	Cas9	1088/17958	Cell proliferation fitness gene	30971826	200-PMID30971826
Lung cancer	NCI-H23	Knock-out	Cas9	36/19875	3D cell proliferation	32238925	5-PMID32238925
Lung cancer	NCI-H23	Knock-out	Cas9	253/19970	3D cell proliferation	32238925	3-PMID32238925
Lung cancer	A549	Knock-out	Cas9	4933/14543	Response to JQ1	31406246	3-PMID31406246
Lymhoma	SU-DHL-4	Knock-out	Cas9	18581/18862	Cell proliferation	28985567	2-PMID28985567
Melanoma	K029AX	Knock-out	Cas9	1359/17670	Cell proliferation essential gene	29083409	131-PMID29083409
Regulatory t cell	CD8^+^ T	Knock-out	Cas9	2475/19108	Cell proliferation	30449619	2-PMID30449619
Urinary bladder cancer	MGH-U4	Knock-out	Cas9	982/16517	Resistance to cisplatin	30414698	1-PMID30414698

### 5.3 The stability link: MiRNAs and HDAC4 in cancer

MicroRNAs (miRNAs) are a class of small non-coding RNAs of approximately 22 nucleotides that regulate gene expression at the post-transcriptional level by repressing translation and/or promoting mRNA degradation (Bartel, 2004). MiRNAs have been shown to be important regulators of a variety of biological processes and diseases, including cancer (Bracken et al., 2016). The list of miRNAs that have been proposed as regulators of HDAC4 is long (Table 4) and a separate review is necessary. Here we will discuss only a few examples.

**TABLE 4:** List of miRNAs influencing HDAC4 levels.

miRNA	Model	Biological process regulated by the miRNA/HDAC4 axis	Reference (doi)
miR-1	Murine myoblasts	Myogenesis	Chen et al. (2006)
	Myoblasts, murine regenerating skeletal muscle	Myogenesis	Sun et al. (2010)
	Growth plate cartilage	Chondrocyte hypertrophy	Li et al. (2014)
	Hepatocellular carcinoma cells	Cancer progression	Datta et al. (2008)
	Human chordoma tissue	Cancer progression	Duan et al. (2010)
miR-9	Neural stem-cells	Neurogenesis	Davila et al. (2014)
	Waldenstrom macroglobuliemia cells	Pathogenesis	Roccaro et al. (2010)
miR-22	Huntington’s and Alzeheimer’s disease brains	Neurodegeneration	Jovicic et al. (2013)
	Murine cardiac tissue	Cardiac hypertrophy	Huang et al. (2013)
	Hepatocellular carcinoma	Cancer progression	Zhang et al. (2010)
	Colon cancer	Cancer progression	He et al. (2019)
	Breast cancer	Cancer progression	Wang et al. (2018)
	Human primary CD4+ T cells, intestinal mucosa tissues	Differentiation of Th17 cells	Pei et al. (2018)
miR-29a	Myogenic C2C12 cell line and primary muscle cells	Myogenesis	Winbanks et al. (2011)
	Murine osteoblasts	Osteoblast differentiation	Ko et al. (2013)
miR-29b	Rat primary osteoblasts, mouse osteoblasts	Osteoblast differentiation	Li et al. (2009)
miR-125a-5p	Human brest cancer	Cancer progression	Hsieh et al. (2015)
	Breast cancer	Cancer aggressiveness	Nishida et al. (2011)
miR-128-3p	Human rheumatoid arthritis fibroblast-like synoviocytes	Rheumatoid arthritis progression	Peng et al. (2021)
miR-140	Human osteosarcoma and colon cancer cells	Chemosensitivity	Song et al. (2009)
miR-141-3p	Human osteoblasts	Osteoblast proliferation and migration	Yu et al. (2022)
miR-145-3p	Human multiple myeloma	Autophagy, cell death, sensitivity to bortezomib	Yu et al. (2022)
miR-145-5p	Human colorectal cancer	Regulation of p53, autophagy	Zhao et al. (2022)
miR-155	Murine B-cells	B-cells proliferation	Sandhu et al. (2012)
miR-200a	Hepatocellular carcinoma cells	Cancer proliferation and migration	Yuan et al. (2011)
miR-206	Myogenic C2C12 cell line and primary muscle cells	Myogenesis	Winbanks et al. (2011)
	Primary tumors	Tumorigenesis	Singh et al. (2013)
	Neuromuscular synapse in mice	Amyotrophic lateral sclerosis	Williams et al. (2009)
	Murine spinal muscular atrophic	Muscle hypertrophy	Valsecchi et al. (2015)
	Gastric tumor	Cancer progression	Ren et al. (2014)
miR-365	Chicken chondrocytes	Chondrocyte hypertrophy	Guan et al. (2011)
	Mouse osteoblasts	Osteogenesis, glucocorticoid-induced osteoporosis	Xu et al. (2017)
miR-378a-3p	Mouse myoblasts	Myoblast differentiation, apoptosis	Wei et al. (2016)
miR-381	Mouse chondrogenic cell line	Chondrogenesis	Chen et al. (2017)
miR-483-5p	Human fetal brain	Neurogenesis	Han et al. (2013)
miR-548ah	Human hepatocarcinoma, Human hepatoblastoma	Replication and expression of Hepatitis B Virus	Xing et al. (2019)

The TF, Nuclear Factor Erythroid-2 Related Factor-2 (NRF2) is the key regulator of the antioxidant response. NRF2 signaling in cancer cells attenuates the expression of miR-1 and miR-206 by promoting the expression of HDAC4, causing a shift in glucose metabolism to the pentose phosphate pathway (PPP) (Singh et al., 2013). HDAC4 in turn represses the expression of miR-1 and miR-206, suggesting that there is a feedback loop between miR-1/miR-206 and HDAC4 that regulates glucose metabolism. In addition, HDAC4 may also function as a redox sensor. In the reduced state, it can repress miR-1/miR-206 transcription and promote PPP gene expression, whereas in the oxidized state, it is sequestered in the cytoplasm, resulting in decreased PPP gene expression.

In hepatocarcinoma cell lines, miR-200a and miR-22 downregulate HDAC4 and their expression negatively correlates with cancer proliferation and migration, suggesting their role as tumor suppressors (Zhang et al., 2010; Yuan et al., 2011). Moreover, HDAC4 mediates transcriptional repression of miR-200a by binding Sp1, resulting in an autoregulatory cycle (Yuan et al., 2011). In colon cancer cells, miR-22 reduces HDAC4 levels, which impairs cancer progression (Hu et al., 2019). The link between miR-22 and HDAC4 has also been confirmed in breast cancer. Downregulation of miR-22 increases HDAC4 levels. In a cellular model of resistance to fulvestrant, a change in miR-22 levels, both an increase and a decrease, affects cell cycle progression, reflecting its impact on multiple targets with antagonistic activities (Wang et al., 2018).

Other miRNAs involved in the control of HDAC4 mRNA stability in cancer cells are miR-145-3p and miR-145-5p. MiR-145-3p is important in limiting multiple myeloma aggressiveness and its overexpression inhibits cell proliferation and autophagy. An activity that synergizes with the action of the proteasome inhibitor bortezomib by enhancing its efficacy (Wu et al., 2020) (see below). miR-145-5p targets HDAC4 and promotes tumor suppressor p53 activation and autophagy activation. Downregulation of miR-145-5p is observed in patients with colorectal cancer (CRC) and is associated with poor prognosis. *In vitro* models of CRC, the transcription factor ATF4 is upregulated and controls the miR-145-5p/HDAC4/p53 axis by inhibiting the expression of miR-145-5p, thereby promoting tumorigenesis, autophagy, and chemoresistance to 5-FU (Zhao et al., 2022a).

In gastric tumors, low expression of miR-206 is associated with better prognosis, suggesting a possible inhibitory role of this miRNA in cancer progression. This tumor suppressive effect is thought to be mediated by inhibition of a variety of target genes, including HDAC4 (Ren et al., 2014). A similar effect is mediated by miR-125a-5p in breast cancer (Hsieh et al., 2015).

Expression of miR-155 in mouse B cells causes proliferation of pre-B cells and high-grade lymphoma or leukemia by suppressing B cell lymphoma-6 (Bcl6) *via* various mechanisms, including HDAC4 downregulation. Indeed, HDAC4 is a co-compressor partner of Bcl6 and a target of miR-155, also a regulatory circuit. In this context, the functions of HDAC4 are tumor suppressive. Indeed, ectopic expression of HDAC4 in diffuse large B cell lymphoma cells of the B cell type leads to reduced miR-155-induced proliferation and induction of cell death (Sandhu et al., 2012).

Various non-coding RNAs can bind competitively to miRNAs, leading to the concept of competing endogenous RNAs (ceRNAs) (Salmena et al., 2011). For example, in the case of HDAC, circ-RNA lipoprotein receptor 6 (circ-LRP6) can abrogate miR-141-3p-mediated repression of HDAC4. circ-LRP6 is highly expressed in osteosarcoma tissues and cell lines (OS), and its expression correlates with cell proliferation and lower overall survival in OS metastatic patients. In contrast, high levels of its target miR-141-3p correlate with higher overall survival (Yu et al., 2022).

Nuclear factor erythroid-2 related factor-2 (NRF2) signaling in cancer cells attenuates miR-1 and miR-206 expression by promoting the expression of *HDAC4*, thus inducing a shift of glucose metabolism towards the pentose phosphate pathway (PPP) (Singh et al., 2013). HDAC4 in turns increases miR-1 and miR-206 expression, suggesting the existence of a feedback loop involving miR-1/miR-206 and HDAC4 that regulates glucose metabolism. In addition, HDAC4 may act also as a redox sensor. In the reduced state it can repress miR-1/miR-206 transcription and promote PPP gene expression, whereas in the oxidized state it is sequestered in the cytoplasm resulting in decreased expression of PPP genes.

In hepatocarcinoma cell lines miR-200a and miR-22 downregulate HDAC4 and their expression negatively correlates with cancer proliferation and migration, suggesting their role as tumor suppressors (Zhang et al., 2010; Yuan et al., 2011). In addition, HDAC4 mediates the transcriptional repression of miR-200a by binding Sp1, creating an autoregulative circuit (Yuan et al., 2011). In colon cancer cells miR-22 reduces HDAC4 levels, affecting the progression of the cancer (Hu et al., 2019).

The relation between miR-22 and HDAC4 was confirmed also in breast cancer, downregulation of miR-22 increases HDAC4 levels. In this cellular model of resistance to fulvestrant altering miR-22 levels, both increasing or decreasing its levels impact on cell cycle progression, thus reflecting its influence on multiple targets with antagonizing activities (Wang et al., 2018).

Additional miRNAs implicated in the control of HDAC4 mRNA stability in cancer cells are miR-145-3p and miR-145-5p. miR-145-3p is important for limiting multiple myeloma aggressiveness and its overexpression inhibits cell proliferation and autophagy, cooperating with the action of the proteasome inhibitor bortezomib and intensifying its efficacy (Wu et al., 2020). miR-145-5p targets HDAC4, promoting the activation of the tumor suppressor p53 and activation of autophagy. Downregulation of miR-145-5p is observed in colorectal cancer (CRC) patients and is related to poor prognosis. *In vitro* models of CRC the transcription factor ATF4 is upregulated and controls the miR-145-5p/HDAC4/p53 axis by inhibiting the expression of miR-145-5p, thus enhancing tumorigenesis, autophagy, and chemoresistance to 5-FU (Zhao L et al., 2022).

In gastric tumor, low expression of miR-206 is associated with a better prognosis, suggesting a potential inhibitory role of this miRNA in cancer progression and this tumor-suppressor action is supposedly mediated by the inhibition of a plethora of target genes, including HDAC4 (Ren et al., 2014). A similar action is mediated by miR-125a-5p in breast cancer (Hsieh et al., 2015).

miR-155 expression in mice B cells causes pre-B cell proliferation and high-grade lymphoma or leukemia by repressing B cell lymphoma-6 (Bcl6) with different mechanisms, including HDAC4 repression. In fact, HDAC4 is a corepressor partner of Bcl6 and it is a target of miR-155, again a regulative circuit. In this context HDAC4 functions are tumor suppressive. In fact, ectopic expression of HDAC4 in B cell-type diffuse large B cell lymphoma cells results in reduced miR-155-induced proliferation and induction of cell death (Sandhu et al., 2012).

Different non-coding RNAs can competitively bind to miRNAs, leading to the concept of competitive endogenous RNAs (ceRNAs) (Salmena et al., 2011). In the case of HDAC, for instance, circ-RNA-lipoprotein receptor 6 (circ-LRP6) can quench the repression mediated by miR-141-3p on HDAC4. circ-LRP6 is highly expressed in osteosarcoma (OS) tissues and cell lines and its expression correlates with cell proliferation and a lower overall survival in OS metastatic patients. On the contrary high levels of its target miR-141-3p, correlate with higher overall survival (Yu et al., 2022).

### 5.4 HDAC4 in hematological malignancies

Among hematological malignancies alterations in HDAC4 has been investigated in Multiple Myeloma (MM). MM is a monoclonal tumor of plasma cells (PCs) that origins from post germinal-center (GC) B cells (Kuehl and Bergsagel, 2012). MM cells are well adapted to endoplasmic reticulum (ER) stress and responsive to drugs that trigger ER and proteotoxic stress. Further increasing the level of proteotoxic stress in MM cells can have a therapeutic benefit (Shah et al., 2015). In MM, overexpression of the chaperone protein BiP and HDAC4 is associated with chemoresistance (Kikuchi S et al., 2015). In this context, the disruption of HDAC4 activity, through both inhibitors and gene silencing, enhanced cytotoxicity induced by inducer of ER-stress (Kikuchi S et al., 2015). Inhibition of HDAC4 potentiates the expression of TFs such as ATF4 and CHOP that can sustain the expression of apoptotic mediators (Brancolini and Iuliano, 2020). Of note, TMP269, a class IIa HDACs selective inhibitor, enhanced the proteotoxic stress and cell death induced by the proteasome inhibitor carfilzomib (Kikuchi S et al., 2015).

Wu et al., confirmed the overexpression of HDAC4 in MM cells and observed, as discussed above, that MIR145-3p inhibits the expression of the deacetylase. Disease progression was associated with the downregulation of miR145-3p and HDAC4 upregulation. Mechanistically, suppression of HDAC4 triggered the upregulation of the pro-apoptotic protein BCL2L11/BIM and caused the inactivation of MTORC1 (Wu H et al., 2020). The authors proposed that alterations in this pathway might enhance autophagy ultimately leading to autophagic cell death. The key role of HDAC4 in MM survival and proliferation was also observed in other studies. Downregulation of HDAC4 triggers the upregulation of miR-29b that controls the levels of the anti-apoptotic BCL2 family member MCL1 (Amodio et al., 2016). The epigenomic surveillance of HDAC4 in MM can be exerted also directly on anti-apoptotic genes. The HDAC4–RelB–p52 complex maintains a repressive status of chromatin around proapoptotic genes such as Bim and BMF (Vallabhapurapu et al., 2015), regulating consequently MM survival and growth (Singh R et al., 2019).

It is important to note that the role of autophagy in tumor cells is complex. At a certain intensity autophagy promotes cell survival in response to metabolic stress, protects against genome damage, limits cell death and inflammation. However, an autophagic boost can lead to cell consumption and autophagic cell death (White and DiPaola, 2009). Moreover, MTORC1 is a key protein complex that regulates not only autophagy, but also cellular growth and proliferation (Saxton and Sabatini, 2017).

In conclusion, it is possible that HDAC4 plays a central role in MM through the regulation of proteostasis and autophagy. Whether this is the central mechanism, or the central mechanism is the epigenetic influence on apoptotic genes cannot be clearly defined. Certainly, the contribution of HDAC4 in several neurodegenerative diseases such as Alzheimer, Dementia and Parkinson—which are well characterized as proteinopathies—supports the proteostasis hypothesis also in tumors (Mielcarek et al., 2015).

As discussed above, epigenetic regulators could play ambiguous roles during tumorigenesis in response to specific environmental cues or genetic alterations. This ambiguity is observed also for HDAC4 in hematological malignancies and particularly in the development of acute myeloid leukemia (AML). In this context, MEF2C acts as an oncogene and sustains cancer aggressiveness and chemoresistance (Di Giorgio et al., 2018). Phosphorylation of HDAC4 through SIK3 (and in part also SIK2) determines cytoplasmic accumulation and disrupts the ability to complex with MEF2C at enhancers sites to buffer H3K27 acetylation. These enhancers regulate cell proliferation, thereby nuclear HDAC4 could inhibit the development of the disease (Tarumoto Y et al., 2018). Given these results, new therapeutic strategies have been explored to increase the nuclear pool of repressive-competent HDAC4. Inhibition of SIK3 activity using the small molecule YKL-05–099 is sufficient to reduce the proliferation of leukemia cells and prolong the survival of a mouse model of AML (MLL-AF9 translocation) (Tarumoto et al., 2020). It is important to note that SIKs have multiple targets and their inhibition might have a pro-proliferative effect where they act as tumor suppressors (Hollstein et al., 2019). A second point that needs to be considered is the possibility of off-target effects of SIK inhibitors (Sakamoto et al., 2018; Darling and Cohen, 2021).

Another tumor suppressive activity of HDAC4, investigated in leukemia cells from AML patients but also observed in various cancer cell lines, provides a link with metabolism. The target is not linked to epigenetic regulation, but it is the cytosolic enzyme 6-phosphogluconate dehydrogenase (6PGD). 6PGD is the third enzyme in the oxidative pentose phosphate pathway (PPP), which catalyzes the decarboxylating reduction of 6-phosphogluconate (6-PG) to ribulose 5-phosphate (Ru-5-P) and produces NADPH in the presence of NADP+ (Shan et al., 2014). Ru-5-P and NADPH can sustain RNA synthesis and lipogenesis and Ru-5-P can also inhibit LKB1–AMPK signaling, all activities that sustain cancer cell proliferation (Lin et al., 2015). HDAC4 reduces the activity of 6PGD by deacetylating lysines 76 and 294. An undefined cytosolic HDAC4 complex seems to be involved in this activity. Unfortunately, this interesting result was not further confirmed or developed.

### 5.5 HDAC4 in solid tumors

Various studies have addressed possible contributions of HDAC4 in development and aggressiveness of different solid cancers. Several different biological activities have been described as target of HDAC4. The epithelial mesenchymal transition (EMT) in cancer cells is an important hallmark of increased motility and chemoresistance (Nieto et al., 2016). In lung cancer cells, low levels of AMP-activated protein kinase (AMPK) enhance glycolysis (Warburg effect) and promote EMT and metastasis, through the action of HDAC4 and HDAC5 (Feng et al., 2020). The contribution of HDAC4 to EMT was observed also in esophageal carcinoma cells (Zeng et al., 2016), but not in other cancer models, suggesting that the contribution of HDAC4 could be lineage specific (Choi et al., 2016). In nasopharyngeal carcinoma HDAC4, in complex with HDAC3-NCOR1, represses transcription from the E-cadherin promoter and augments the expression of the mesenchymal markers such as N-cadherin, Snail and Slug (Cheng et al., 2021). The role of HDAC4 in EMT and in the metastatic process required further studies since also anti-metastatic effects have been observed. ZEB1 is an important TF involved in the EMT: it favors the metastatic process by increasing the levels of integrins and the adhesion to collagen fibers. The upregulation of these activities requires the cytoplasmic accumulation of HDAC4 through the inhibition of PP2A (Paroni et al., 2007) to release the epigenetic repression on integrin gene *Itga1* (Tan et al., 2022). An anti-metastatic role of HDAC4 was also observed in ovarian cancer and is operated by the classical partner MEF2A. Mechanistically, the phosphatase PRL-3 binds HDAC4 and competes for the binding to MEF2A, which in turn is released and can promote acetylation and transcription at the SOX2 locus. This circuit allows the expansion of cancer stem cells, tumor aggressiveness and metastasis (Zhang et al., 2020). This result is in agreement with the possible involvement of MEF2 family members in EMT (Su et al., 2016; Xiao L et al., 2021).

The increased proliferative capacity of tumors is due to alterations in the normal regulation of the cell cycle, and the contribution of HDAC4 to cell cycle progression has been observed in various contexts, including normal cells and different cancer models (Wilson et al., 2008; Feng et al., 2009; Cai et al., 2018; Bindea et al., 2009; Cao et al., 2019; Ha et al., 2022). Experiments in some reports have demonstrated that the expression of CDK inhibitors (CDKN1A and CDKN2A) is regulated in an HDAC4-dependent manner. Overall, the mechanisms used by HDAC4 to influence cell cycle progression deserve further analysis. In particular, the protein complexes monitored by HDAC4 that epigenetically control transcription of these inhibitors have not been clearly elucidated. Recently, the tumor suppressor gene breast cancer type 1 BRCA1-associated protein 1 (BAP1) was shown to antagonize HDAC4 activity to reduce proliferation. BAP1 encodes a deubiquitylase that regulates the level of ubiquitylation of histone H2AK119, a repressive epigenetic mark involved in the regulation of transcription, replication, and repair. Uveal melanoma (UM) and malignant pleural mesothelioma (MPM) are among the tumors with the highest incidence of BAP1 mutations. In BAP1-mutated UM cells, HDAC4 is upregulated, accumulates in nuclei, and is required for cell proliferation. HDAC4 controls the expression of lineage-specific genes by reducing the level of H3K27 acetylation (Kuznetsov et al., 2019). The link between BAP1 and HDAC4 may also provide a strategy for personalized therapeutic intervention, as recently proposed (Kuznetsoff et al., 2021).

Cellular senescence is a response to various cellular stresses that leads to irreversible arrest of the cell cycle and secretion of inflammatory and regenerative cytokines (Bousset and Gil, 2022). Oncogenic lesions, by enhancing cell proliferation, lead to accumulation of DNA damage and induction of senescence, also known as oncogene-induced senescence (OIS). OIS can limit the tumorigenic process and eventually promotes the elimination of tumor cells by the immune system. Senescence escape has been recognized as one of the hallmarks of cancer cells (Hanahan et al., 2022). Conversely, induction of senescence may promote tumor recurrence under certain circumstances, for example, in response to chemotherapy (Demaria et al., 2017). HDAC4 is downregulated with aging and during various forms of senescence by UPS in a GSK3β-dependent manner (Han et al., 2016; Di Giorgio et al., 2021; Lee et al., 2022). The anti-senescence effect of HDAC4 appears to be exploited on multiple levels. Directly or indirectly, HDAC4 may reduce the transcription of CDK inhibitors, the major brakes of the cell cycle machinery that are upregulated in senescent cells (Wilson et al., 2008; Feng et al., 2009; Wang et al., 2012), or other genes of the senescence program (Lee et al., 2022). An important epigenetic strategy is employed by HDAC4 to antagonize OIS. HDAC4 can buffer the activation of typical enhancers and super-enhancers activated during senescence (Di Giorgio et al., 2021). Super-enhancers (SEs) are important regulatory domains of chromatin that span several kilobases and have a high density of TFs and other regulators of transcription to effectively control gene expression (Hnisz et al., 2017). In this activity, HDAC4 requires the action of HDAC3 to limit H3K27 acetylation. Interestingly, several of these SEs that control senescence are activated by the AP-1 TFs, in complex with the HAT p300/KAT3B (Martínez-Zamudio et al., 2020).

Similar to senescence, cell death by apoptosis is an important antiproliferative option both in terms of limiting tumor growth and eradicating it in response to therapy. As discussed above, in absence of HDAC4 MM cells are more prone to proteotoxic stress-induced cell death, possibly indicating an augmentation of the normal level of protein misfolding (Kikuchi et al., 2015). This effect could explain the increased susceptibility to TRAIL-induced cell death observed in cells deficient in HDAC4 (Lee et al., 2018). The pro-survival activities of HDAC4 could also be related to modulation of DNA damage repair. In hepatocellular carcinoma, HDAC4 contributes to the efficiency of double strand brakes (DSBs) repair, by sustaining the homologous recombination. Repair of DSBs occurs *via* two main pathways: Non-homologous end joining (NHEJ) and homologous recombination (HR). Alternative EJ repair (Alt-EJ) mechanisms may also be employed (Ceccaldi et al., 2016). In this context HDAC4 contributes to the nuclear accumulation of RAD51 (Tsai et al., 2018), a key element of the machinery for repairing DSBs (Anand et al., 2017). A possible, but not yet clearly defined, role of HDAC4 in DSBs repair has also been observed by others (Marampon et al., 2017). In pancreatic cancer cells, the serin-threonine kinase MARK2 is activated in response to paclitaxel treatment, a microtubule disrupting agent. HDAC4 is a substrate of MARK2 and once phosphorylated promotes the activity of YAP and resistance to paclitaxel. (Zeng et al., 2022). In some cases, a direct intervention of HDAC4 in the regulation of apoptotic programs has been reported. HDAC4 can repress transcription of core elements of the apoptotic machinery, such as BMF (Vallabhapurapu et al., 2015). The pro-survival activity of HDAC4 can also be exploited by controlling the tumor microenvironment. IL24 is a cytokine that can kill cancer cells and several attempts have been made to design a therapeutic approach using this cytokine (Menezes et al., 2014). HDAC4 can repress transcription of IL24 through epigenetic control at the promoter level (Pan et al., 2010), a property it shares with other class IIa HDACs (Cutano et al., 2019). In conclusion, although there are several data confirming a role of HDAC4 in cancer cell survival, the mechanism seems to vary from case to case. We should always consider a possible redundancy with other members of the family and the engagement of compensatory mechanisms, two conditions that complicate the interpretation of the data and could explain the heterogeneity of the results obtained. Certainly, interfering with HDAC4 levels using RNAi, increases cancer cell susceptibility to different apoptotic stimuli or cellular stressors. For this reason, several attempts have been made to affect the activity of HDAC4, by isoform-specific inhibitors.

Another characteristic of cancer cells is their ability to adapt to an unfavorable metabolic environment. This hurdle can be circumvented by metabolic adaptation to oxygen deprivation or/and induction of angiogenesis, and both have been reported to be regulated by HDAC4. The transcription factor HIF1 is the master regulator of oxygen homeostasis and consists of the subunits HIF1A and HIF1B. Under oxygen-rich conditions, HIF1A is constantly degraded *via* UPS by the action of Von Hippel-Lindau protein (VHL), which promotes the action of an E3 ubiquitin ligase. Hypoxic conditions selectively suppress this degradation because the absence of prolyl and asparaginyl hydroxylation in HIF1A at critical residues inhibits recognition by the VHL protein (Semenza, 2012; Semenza, 2016). An interaction between HIF1A and HDAC4 has been reported to affect the transcriptional activity of HIF1. The mechanism appears to involve HDAC4-dependent deacetylation and stabilization of HIF1A with potentiation of its transcriptional activity (Qian et al., 2006; Geng et al., 2011; Fischer et al., 2015; Chen et al., 2016). Alternative mechanisms have also been proposed (Seo et al., 2009; Chen et al., 2016). Recently, Zhang et al. (2017) showed that Nucleus Accumbens Associated 1 (NAC1) binds to HDAC4 and prevents its cytoplasmic degradation, enhancing the adaptive response to hypoxia. On the other hand, Fan et al. (2021) have shown that HIF and VEGFA are functional downstream mediators of HDAC4 *via* the ZIP4-HDAC4-VEGFA axis in high-grade serous ovarian cancer. These two features are not necessarily distinct and can be exploited in a potential therapeutic horizon. In a “synthetic lethality” scenario (Huang et al., 2020), the combination of blocking HDAC4 and using monoclonal antibodies against VEGF, such as bevacizumab, may be promising in the overwhelming landscape of tumor resistance, and some encouraging results have already been obtained (Zhang et al., 2017).

## 6 Conclusion

More than two decades after its discovery, there are still many puzzling aspects and unanswered questions about HDAC4. Although its role as a repressor of MEF2 and modulator of the epigenome is well established, experimental data suggest that alternative targeting to different genomic regions is also possible, independently of MEF2. The composition of these MEF2-independent complexes is not known. Whether the other TFs regulated by HDAC4 may also contribute to localize HDAC4-coordinated complexes to other genomic regions remains to be investigated. Another point of interest is the dynamic balance between HDAC4 acting as an epigenomic regulator and HDAC4 complexes targeting non-histone proteins. The cytoplasmic functions of HDAC4 are also poorly understood, and it is still unclear whether the composition of HDAC4 complexes formed in the cytosol differs from complexes formed in the nucleus. Although the pro-oncogenic role of HDAC4 in cancer is known, conditions under which HDAC4 activities antagonize the transformation process are conceivable. Although cancer genome projects have allowed us to explore the degree of dysregulation of several genes, including HDAC4 in tumors, we have yet to gain information about the molecular complexity of the protein and its partners.

## References

[B1] Aloia L. (2022). Epigenetic regulation of cell-fate changes that determine adult liver regeneration after injury. Front. Cell Dev. Biol. 9, 643055. 10.3389/fcell.2021.643055 PMC7957008 33732709

[B2] Amodio N. Stamato M. A. Gullà A. M. Morelli E. Romeo E. Raimondi L. (2016). Therapeutic targeting of miR-29b/HDAC4 epigenetic loop in multiple myeloma. Mol. Cancer Ther. 15 (6), 1364–1375. 10.1158/1535-7163.MCT-15-0985 27196750

[B3] Anand R. Beach A. Li K. Haber J. (2017). Rad51-mediated double-strand break repair and mismatch correction of divergent substrates. Nature 544 (7650), 377–380. 10.1038/nature22046 28405019 PMC5544500

[B4] Assali A. Harrington A. J. Cowan C. W. (2019). Emerging roles for MEF2 in brain development and mental disorders. Curr. Opin. Neurobiol. 59, 49–58. 10.1016/j.conb.2019.04.008 31129473 PMC6874740

[B5] Backs J. Song K. Bezprozvannaya S. Chang S. Olson E. N. (2006). CaM kinase II selectively signals to histone deacetylase 4 during cardiomyocyte hypertrophy. J. Clin. Invest. 116 (7), 1853–1864. 10.1172/JCI27438 16767219 PMC1474817

[B225] Backs J. Worst B. C. Lehmann L. H. Patrick D. M. Jebessa Z. Kreusse M. M. (2011). Selective repression of MEF_2_ activity by PKA-dependent proteolysis of HDAC_4_ . J. Cell Biol. 195 (3), 403–415. 10.1083/jcb.201105063 22042619 PMC3206346

[B6] Bartel D. P. (2004). MicroRNAs: Genomics, biogenesis, mechanism, and function. Cell 116 (2), 281–297. 10.1016/s0092-8674(04)00045-5 14744438

[B7] Berdeaux R. Goebel N. Banaszynski L. Takemori H. Wandless T. Shelton G. D. (2007). SIK1 is a class II HDAC kinase that promotes survival of skeletal myocytes. Nat. Med. 13 (5), 597–603. 10.1038/nm1573 17468767

[B223] Bindea G. Mlecnik B. Hackl H. Charoentong P. Tosolini M. Kirilovsky A. (2009). ClueGO: A Cytoscape plug-in to decipher functionally grouped gene ontology and pathway annotation networks. Bioinformatics 25 (8), 1091–1093. 10.1093/bioinformatics/btp101 19237447 PMC2666812

[B8] Bodily J. M. Mehta K. P. Laimins L. A. (2011). Human papillomavirus E7 enhances hypoxia-inducible factor 1-mediated transcription by inhibiting binding of histone deacetylases. Cancer Res. 71 (3), 1187–1195. 10.1158/0008-5472.CAN-10-2626 21148070 PMC3077548

[B9] Bottomley M. J. Lo Surdo P. Di Giovine P. Cirillo A. Scarpelli R. Ferrigno F. (2008). Structural and functional analysis of the human HDAC4 catalytic domain reveals a regulatory structural zinc-binding domain. J. Biol. Chem. 283 (39), 26694–26704. 10.1074/jbc.M803514200 18614528 PMC3258910

[B10] Bousset L. Gil J. (2022). Targeting senescence as an anticancer therapy. Mol. Oncol. 16 (21), 3855–3880. 10.1002/1878-0261.13312 36065138 PMC9627790

[B11] Bracken C. P. Scott H. S. Goodall G. J. (2016). A network-biology perspective of microRNA function and dysfunction in cancer. Nat. Rev. Genet. 17 (12), 719–732. 10.1038/nrg.2016.134 27795564

[B12] Brancolini C. Di Giorgio E. Formisano L. Gagliano T. (2021). Quis custodiet ipsos custodes (who controls the controllers)? Two decades of studies on HDAC9. Life (Basel) 27.11 (2), 90. 10.3390/life11020090 PMC7912504 33513699

[B13] Brancolini C. Gagliano T. Minisini M. (2022). HDACs and the epigenetic plasticity of cancer cells: Target the complexity. Pharmacol. Ther. 238, 108190. 10.1016/j.pharmthera.2022.108190 35430294

[B14] Brancolini C. Iuliano L. (2020). Proteotoxic stress and cell death in cancer cells. Cancers (Basel) 12 (9), 2385. 10.3390/cancers12092385 32842524 PMC7563887

[B15] Cadot B. Brunetti M. Coppari S. Fedeli S. de Rinaldis E. Dello Russo C. (2009). Loss of histone deacetylase 4 causes segregation defects during mitosis of p53-deficient human tumor cells. Cancer Res. 69 (15), 6074–6082. 10.1158/0008-5472.CAN-08-2796 19622775

[B16] Cai J. Y. Xu T. T. Wang Y. Chang J. J. Li J. Chen X. Y. (2018). Histone deacetylase HDAC4 promotes the proliferation and invasion of glioma cells. Int. J. Oncol. 53 (6), 2758–2768. 10.3892/ijo.2018.4564 30272277

[B17] Cao K. Wang H. Fang Y. Wang Y. Wei L. Chen X. (2019). Histone deacetylase 4 promotes osteosarcoma cell proliferation and invasion by regulating expression of proliferating cell nuclear antigen. Front. Oncol. 9, 870. 10.3389/fonc.2019.00870 31552187 PMC6743440

[B18] Cao K. Wei L. Zhang Z. Guo L. Zhang C. Li Y. (2014). Decreased histone deacetylase 4 is associated with human osteoarthritis cartilage degeneration by releasing histone deacetylase 4 inhibition of runt-related transcription factor-2 and increasing osteoarthritis-related genes: A novel mechanism of human osteoarthritis cartilage degeneration. Arthritis Res. Ther. 16 (6), 491. 10.1186/s13075-014-0491-3 25424126 PMC4265470

[B19] Ceccaldi R. Rondinelli B. D'Andrea A. D. (2016). Repair pathway choices and consequences at the double-strand break. Trends Cell Biol. 26 (1), 52–64. 10.1016/j.tcb.2015.07.009 26437586 PMC4862604

[B20] Cernotta N. Clocchiatti A. Florean C. Brancolini C. (2011). Ubiquitin-dependent degradation of HDAC4, a new regulator of random cell motility. Mol. Biol. Cell 22 (2), 278–289. 10.1091/mbc.E10-07-0616 21118993 PMC3020922

[B21] Chen J. Mandel E. M. Thomson J. M. Wu Q. Callis T. E. Hammond S. M. (2006). The role of microRNA-1 and microRNA-133 in skeletal muscle proliferation and differentiation. Nat. Genet. 38 (2), 228–233. 10.1038/ng1725 16380711 PMC2538576

[B22] Chen S. Sang N. (2016). Hypoxia-inducible factor-1: A critical player in the survival strategy of stressed cells. J. Cell Biochem. 117 (2), 267–278. 10.1002/jcb.25283 26206147 PMC4715696

[B23] Chen W. Sheng P. Huang Z. Meng F. Kang Y. Huang G. (2016). MicroRNA-381 regulates chondrocyte hypertrophy by inhibiting histone deacetylase 4 expression. Int. J. Sci. 17 (9), 1377. 10.3390/ijms17091377 PMC5037657 27563877

[B24] Chen Z. Zhang Z. Guo L. Wei X. Zhang Y. Wang X. (2020). The role of histone deacetylase 4 during chondrocyte hypertrophy and endochondral bone development. Bone Jt. Res. 9 (2), 82–89. 10.1302/2046-3758.92.BJR-2019-0172.R1 PMC7229302 32435460

[B25] Cheng C. Yang J. Li S. W. Huang G. Li C. Min W. P. (2021). HDAC4 promotes nasopharyngeal carcinoma progression and serves as a therapeutic target. Cell Death Dis. 12 (2), 137. 10.1038/s41419-021-03417-0 33542203 PMC7862285

[B26] Choi M. C. Ryu S. Hao R. Wang B. Kapur M. Fan C. M. (2014). HDAC4 promotes Pax7-dependent satellite cell activation and muscle regeneration. EMBO Rep. 15 (11), 1175–1183. 10.15252/embr.201439195 25205686 PMC4253491

[B27] Choi S. Y. Kee H. J. Kurz T. Hansen F. K. Ryu Y. Kim G. R. (2016). Class I HDACs specifically regulate E-cadherin expression in human renal epithelial cells. J. Cell Mol. Med. 20 (12), 2289–2298. 10.1111/jcmm.12919 27420561 PMC5134402

[B28] Clocchiatti A. Di Giorgio E. Viviani G. Streuli C. Sgorbissa A. Picco R. (2015). The MEF2-HDAC axis controls proliferation of mammary epithelial cells and acini formation *in vitro* . J. Cell Sci. 128 (21), 3961–3976. 10.1242/jcs.170357 26403201

[B29] Clocchiatti A. Di Giorgio E. Demarchi F. Brancolini C. (2013). Beside the MEF2 axis: Unconventional functions of HDAC4. Cell Signal 25 (1), 269–276. 10.1016/j.cellsig.2012.10.002 23063464

[B30] Cutano V. Di Giorgio E. Minisini M. Picco R. Dalla E. Brancolini C. (2019). HDAC7-mediated control of tumour microenvironment maintains proliferative and stemness competence of human mammary epithelial cells. Mol. Oncol. 13 (8), 1651–1668. 10.1002/1878-0261.12503 31081251 PMC6670296

[B31] Darling N. J. Cohen P. (2021). Nuts and bolts of the salt-inducible kinases (SIKs). Biochem. J. 478 (7), 1377–1397. 10.1042/BCJ20200502 33861845 PMC8057676

[B32] Datta J. Kutay H. Nasser M. W. Nuovo G. J. Wang B. Majumder S. (2008). Methylation mediated silencing of microRNA-1 gene and its role in hepatocellular carcinogenesis. Cancer Res. 68 (13), 5049–5058. 10.1158/0008-5472.CAN-07-6655 18593903 PMC2562630

[B33] Davila J. L. Goff L. A. Ricupero C. L. Camarillo C. Oni E. N. Swerdel M. R. (2014). A positive feedback mechanism that regulates expression of miR-9 during neurogenesis. PLoS One 9 (4), e94348. 10.1371/journal.pone.0094348 24714615 PMC3979806

[B34] Dehennaut V. Loison I. Dubuissez M. Nassour J. Abbadie C. Leprince D. (2013). DNA double-strand breaks lead to activation of hypermethylated in cancer 1 (HIC1) by SUMOylation to regulate DNA repair. J. Biol. Chem. 288 (15), 10254–10264. 10.1074/jbc.M112.421610 23417673 PMC3624409

[B35] Demaria M. O'Leary M. N. Chang J. Shao L. Liu S. Alimirah F. (2017). Cellular senescence promotes adverse effects of chemotherapy and cancer relapse. Cancer Discov. 7 (2), 165–176. 10.1158/2159-8290.CD-16-0241 27979832 PMC5296251

[B36] Dequiedt F. Martin M. Von Blume J. Vertommen D. Lecomte E. Mari N. (2006). New role for hPar-1 kinases EMK and C-TAK1 in regulating localization and activity of class IIa histone deacetylases. Mol. Cell Biol. 26 (19), 7086–7102. 10.1128/MCB.00231-06 16980613 PMC1592903

[B37] Di Giorgio E. Brancolini C. (2016). Regulation of class IIa HDAC activities: It is not only matter of subcellular localization. Epigenomics 8 (2), 251–269. 10.2217/epi.15.106 26791815

[B38] Di Giorgio E. Clocchiatti A. Piccinin S. Sgorbissa A. Viviani G. Peruzzo P. (2013). MEF2 is a converging hub for histone deacetylase 4 and phosphatidylinositol 3-kinase/Akt-induced transformation. Mol. Cell Biol. 33 (22), 4473–4491. 10.1128/MCB.01050-13 24043307 PMC3838174

[B39] Di Giorgio E. Dalla E. Franforte E. Paluvai H. Minisini M. Trevisanut M. (2020). Different class IIa HDACs repressive complexes regulate specific epigenetic responses related to cell survival in leiomyosarcoma cells. Nucleic Acids Res. 48 (2), 646–664. 10.1093/nar/gkz1120 31754707 PMC6954409

[B40] Di Giorgio E. Franforte E. Cefalù S. Rossi S. Dei Tos A. P. Brenca M. (2017). The co-existence of transcriptional activator and transcriptional repressor MEF2 complexes influences tumor aggressiveness. PLoS Genet. 13 (4), e1006752. 10.1371/journal.pgen.1006752 28419090 PMC5413110

[B41] Di Giorgio E. Gagliostro E. Brancolini C. (2015). Selective class IIa HDAC inhibitors: Myth or reality. Cell Mol. Life Sci. 72 (1), 73–86. 10.1007/s00018-014-1727-8 25189628 PMC11113455

[B42] Di Giorgio E. Hancock W. W. Brancolini C. (2018). MEF2 and the tumorigenic process, hic sunt leones. Biochim. Biophys. Acta Rev. Cancer 1870 (2), 261–273. 10.1016/j.bbcan.2018.05.007 29879430

[B43] Di Giorgio E. Paluvai H. Dalla E. Ranzino L. Renzini A. Moresi V. (2021). HDAC4 degradation during senescence unleashes an epigenetic program driven by AP-1/p300 at selected enhancers and super-enhancers. Genome Biol. 22 (1), 129. 10.1186/s13059-021-02340-z 33966634 PMC8108360

[B44] Ding Y. Xing D. Fei Y. Lu B. (2022). Emerging degrader technologies engaging lysosomal pathways. Chem. Soc. Rev. 51 (21), 8832–8876. 10.1039/d2cs00624c 36218065 PMC9620493

[B45] Doddi S. K. Kummari G. Jagannadham M. V. Kalle A. M. (2019). Protein kinase A mediates novel serine-584 phosphorylation of HDAC4. Biochem. Cell Biol. 97 (5), 526–535. 10.1139/bcb-2018-0208 30661366

[B46] Du J. Zhang L. Zhuang S. Qin G. J. Zhao T. C. (2015). HDAC4 degradation mediates HDAC inhibition-induced protective effects against hypoxia/reoxygenation injury. J. Cell Physiol. 230 (6), 1321–1331. 10.1002/jcp.24871 25475100 PMC4373665

[B47] Duan Z. Choy E. Nielsen G. P. Rosenber A. Iafrate J. Yang C. (2010). Differential expression of microRNA (miRNA) in chordoma reveals a role for miRNA-1 in Met expression. J. Orthop. Res. 28 (6), 746–752. 10.1002/jor.21055 20041488

[B48] Fan Q. Li L. Wang T. L. Emerson R. E. Xu Y. (2021). A novel ZIP4-HDAC4-VEGFA Axis in high-grade serous ovarian cancer. Cancers (Basel) 13 (15), 3821. 10.3390/cancers13153821 34359722 PMC8345154

[B49] Feng S. Zhang L. Liu X. Li G. Zhang B. Wang Z. (2020). Low levels of AMPK promote epithelial-mesenchymal transition in lung cancer primarily through HDAC4- and HDAC5-mediated metabolic reprogramming. J. Cell Mol. Med. 24 (14), 7789–7801. 10.1111/jcmm.15410 32519437 PMC7348170

[B50] Feng Y. Wang X. Xu L. Pan H. Zhu S. Liang Q. (2009). The transcription factor ZBP-89 suppresses p16 expression through a histone modification mechanism to affect cell senescence. FEBS J. 276 (15), 4197–4206. 10.1111/j.1742-4658.2009.07128.x 19583777

[B51] Finke D. Schanze L. M. Schreiter F. Kreußer M. M. Katus H. A. Backs J. (2022). Histone deacetylase 4 deletion broadly affects cardiac epigenetic repression and regulates transcriptional susceptibility via H3K9 methylation. J. Mol. Cell Cardiol. 162, 119–129. 10.1016/j.yjmcc.2021.09.001 34492228

[B52] Fischer C. Leithner K. Wohlkoenig C. Quehenberger F. Bertsch A. Olschewski A. (2015). Panobinostat reduces hypoxia-induced cisplatin resistance of non-small cell lung carcinoma cells via HIF-1α destabilization. Mol. Cancer 14, 4. 10.1186/1476-4598-14-4 25608569 PMC4320451

[B53] Franklin K. A. Shields C. E. Haynes K. A. (2022). Beyond the marks: Reader-effectors as drivers of epigenetics and chromatin engineering. Trends Biochem. Sci. 47 (5), 417–432. 10.1016/j.tibs.2022.03.002 35427480 PMC9074927

[B54] Geng H. Harvey C. T. Pittsenbarger J. Liu Q. Beer T. M. Xue C. (2011). HDAC4 protein regulates HIF1α protein lysine acetylation and cancer cell response to hypoxia. J. Biol. Chem. 286 (44), 38095–38102. 10.1074/jbc.M111.257055 21917920 PMC3207467

[B55] Gil V. S. Bhagat G. Howell L. Zhang J. Kim C. H. Stengel S. (2016). Deregulated expression of HDAC9 in B cells promotes development of lymphoproliferative disease and lymphoma in mice. Dis. Model Mech. 9 (12), 1483–1495. 10.1242/dmm.023366 27799148 PMC5200892

[B56] Greco T. M. Yu F. Guise A. J. Cristea I. M. (2010). Nuclear import of histone deacetylase 5 by requisite nuclear localization signal phosphorylation. Mol. Cell Proteomics 10 (2), M110.004317. 10.1074/mcp.M110.004317 PMC3033682 21081666

[B57] Grégoire S. Tremblay A. M. Xiao L. Yang Q. Ma K. Nie J. (2006). Control of MEF2 transcriptional activity by coordinated phosphorylation and sumoylation. J. Biol. Chem. 281 (7), 4423–4433. 10.1074/jbc.M509471200 16356933

[B58] Griffin E. A. Jr Melas P. A. Zhou R. Li Y. Mercado P. Kempadoo K. A. (2017). Prior alcohol use enhances vulnerability to compulsive cocaine self-administration by promoting degradation of HDAC4 and HDAC5. Sci. Adv. 3 (11), e1701682. 10.1126/sciadv.1701682 29109977 PMC5665598

[B59] Grozinger C. M. Schreiber S. L. (2000). Regulation of histone deacetylase 4 and 5 and transcriptional activity by 14-3-3-dependent cellular localization. Proc. Natl. Acad. Sci. U. S. A. 97 (14), 7835–7840. 10.1073/pnas.140199597 10869435 PMC16631

[B60] Guan Y. J. Yang X. Wei L. Chen Q. (2011). MiR-365: A mechanosensitive microRNA stimulates chondrocyte differentiation through targeting histone deacetylase 4. Faseb J. 25 (12), 4457–4466. 10.1096/fj.11-185132 21856783 PMC3236620

[B61] Guenther M. G. Barak O. Lazar M. A. (2001). The SMRT and N-CoR corepressors are activating cofactors for histone deacetylase 3. Mol. Cell Biol. 21 (18), 6091–6101. 10.1128/MCB.21.18.6091-6101.2001 11509652 PMC87326

[B62] Guise A. J. Greco T. M. Zhang I. Y. Yu F. Cristea I. M. (2012). Aurora B-dependent regulation of class IIa histone deacetylases by mitotic nuclear localization signal phosphorylation. Mol. Cell Proteomics 11 (11), 1220–1229. 10.1074/mcp.M112.021030 22865920 PMC3494195

[B63] Guo L. Han A. Bates D. L. Cao J. Chen L. (2007). Crystal structure of a conserved N-terminal domain of histone deacetylase 4 reveals functional insights into glutamine-rich domains. Proc. Natl. Acad. Sci. U. S. A. 104 (11), 4297–4302. 10.1073/pnas.0608041104 17360518 PMC1838596

[B64] Ha N. Sun J. Bian Q. Wu D. Wang X. (2022). *Hdac4* regulates the proliferation of neural crest-derived osteoblasts during murine craniofacial development. Front. Physiol. 13, 819619. 10.3389/fphys.2022.819619 35242053 PMC8886889

[B65] Han K. Gennarino V. A. Lee Y. Pang K. Hashimoto-Torii K. Choufani S. (2013). Human-specific regulation of MeCP2 levels in fetal brains by microRNA miR-483-5p. Genes Dev. 27 (5), 485–490. 10.1101/gad.207456.112 23431031 PMC3605462

[B66] Hanahan D. (2022). Hallmarks of cancer: New dimensions. Cancer Discov. 12 (1), 31–46. 10.1158/2159-8290.CD-21-1059 35022204

[B67] HanNiu X, J. Zhao Y. Kong Q. Tong T. Han L. (2016). HDAC4 stabilizes SIRT1 via sumoylation SIRT1 to delay cellular senescence. Clin. Exp. Pharmacol. Physiol. 43 (1), 41–46. 10.1111/1440-1681.12496 26414199

[B68] Helmstadter K. G. Ljubojevic-Holzer S. Wood B. M. Taheri K. D. Sedej S. Erickson J. R. (2021). CaMKII and PKA-dependent phosphorylation co-regulate nuclear localization of HDAC4 in adult cardiomyocytes. Basic Res. Cardiol. 116 (1), 11. 10.1007/s00395-021-00850-2 33590335 PMC7884572

[B69] Hnisz D. Shrinivas K. Young R. A. Chakraborty A. K. Sharp P. A. (2017). A phase separation model for transcriptional control. Cell 169 (1), 13–23. 10.1016/j.cell.2017.02.007 28340338 PMC5432200

[B70] Hohl M. Wagner M. Reil J. C. Müller S. A. Tauchnitz M. Zimmer A. M. (2013). HDAC4 controls histone methylation in response to elevated cardiac load. J. Clin. Invest. 123 (3), 1359–1370. 10.1172/JCI61084 23434587 PMC3582114

[B71] Hollstein P. E. Eichner L. J. Brun S. N. Kamireddy A. Svensson R. U. Vera L. I. (2019). The AMPK-related kinases SIK1 and SIK3 mediate key tumor-suppressive effects of LKB1 in NSCLC. Cancer Discov. 9 (11), 1606–1627. 10.1158/2159-8290.CD-18-1261 31350328 PMC6825547

[B72] Hosokawa H. Rothenberg E. V. (2021). How transcription factors drive choice of the T cell fate. Nat. Rev. Immunol. 21 (3), 162–176. 10.1038/s41577-020-00426-6 2doi:32918063 PMC7933071

[B73] Hsieh T. H. Hsu C. Y. Tsai C. F. Long C. Y. Chai C. Y. Hou M. F. (2015). MiR-125a-5p is a prognostic biomarker that targets HDAC4 to suppress breast tumorigenesis. Oncotarget 6 (1), 494–509. 10.18632/oncotarget.2674 25504437 PMC4381610

[B74] Hu Y. French S. W. Chau T. Liu H. X. Sheng L. Wei F. (2019). RARβ acts as both an upstream regulator and downstream effector of miR-22, which epigenetically regulates NUR77 to induce apoptosis of colon cancer cells. Faseb J. 33 (2), 2314–2326. 10.1096/fj.201801390R 30252536 PMC6338632

[B75] Huang A. Garraway L. A. Ashworth A. Weber B. (2020). Synthetic lethality as an engine for cancer drug target discovery. Nat. Rev. Drug Discov. 19 (1), 23–38. 10.1038/s41573-019-0046-z 31712683

[B76] Huang Z. P. Chen J. Seok H. Y. Zhang Z. Kataoka M. Hu X. (2013). MicroRNA-22 regulates cardiac hypertrophy and remodeling in response to stress. Circ. Res. 112 (9), 1234–1243. 10.1161/CIRCRESAHA.112.300682 23524588 PMC3720677

[B77] Hudson G. M. Watson P. J. Fairall L. Jamieson A. G. Schwabe J. W. R. (2015). Insights into the recruitment of class IIa histone deacetylases (HDACs) to the SMRT/NCoR transcriptional repression complex. J. Biol. Chem. 290 (29), 18237–18244. 10.1074/jbc.M115.661058 26055705 PMC4505066

[B78] Jebessa Z. H. Shanmukha K. D. Dewenter M. Lehmann L. H. Xu C. Schreiter F. (2019). The lipid droplet-associated protein ABHD5 protects the heart through proteolysis of HDAC4. Nat. Metab. 1 (11), 1157–1167. 10.1038/s42255-019-0138-4 31742248 PMC6861130

[B226] Jiang H. Jia D. Zhang B. Yang W. Dong Z. Sun X. (2020). Exercise improves cardiac function and glucose metabolism in mice with experimental myocardial infarction through inhibiting HDAC4 and upregulating GLUT1 expression. Basic Res. Cardiol. 115 (3), 28. 10.1007/s00395-020-0787-1 32236769

[B79] Jovicic A. Zaldivar Jolissant J. F. Moser R. Silva Santos M. d. F. Luthi-Carter R. (2013). MicroRNA-22 (miR-22) overexpression is neuroprotective via general anti-apoptotic effects and may also target specific Huntington's disease-related mechanisms. PLoS One 8 (1), e54222. 10.1371/journal.pone.0054222 23349832 PMC3547907

[B80] Jumper J. Evans R. Pritzel A. Green T. Figurnov M. Ronneberger O. (2021). Highly accurate protein structure prediction with AlphaFold. Nature 596 (7873), 583–589. 10.1038/s41586-021-03819-2 34265844 PMC8371605

[B81] Kalaora S. Nagler A. Wargo J. A. Samuels Y. (2022). Mechanisms of immune activation and regulation: Lessons from melanoma. Nat. Rev. Cancer 22 (4), 195–207. 10.1038/s41568-022-00442-9 35105962

[B82] Kang Z. H. Wang C. Y. Zhang W. L. Zhang J. T. Yuan C. H. Zhao P. W. (2014). Histone deacetylase HDAC4 promotes gastric cancer SGC-7901 cells progression via p21 repression. PLoS One 9 (6), e98894. 10.1371/journal.pone.0098894 24896240 PMC4045860

[B83] Kikuchi S. Suzuki R. Ohguchi H. Yoshida Y. Lu D. Cottini F. (2015). Class IIa HDAC inhibition enhances ER stress-mediated cell death in multiple myeloma. Leukemia 29, 1918–1927. 10.1038/leu.2015.83 25801913

[B84] Kim G. R. Cho S. N. Kim H. S. Yu S. Y. Choi S. Y. Ryu Y. (2016). Histone deacetylase and GATA-binding factor 6 regulate arterial remodeling in angiotensin II-induced hypertension. J. Hypertens. 34 (11), 2206–2219. 10.1097/HJH.0000000000001081 27512969

[B85] Kim G. S. Jung H. E. Kim J. S. Lee Y. C. (2015). Mutagenesis study reveals the rim of catalytic entry site of HDAC4 and -5 as the major binding surface of SMRT corepressor. PLoS One 10 (7), e0132680. 10.1371/journal.pone.0132680 26161557 PMC4498904

[B86] Kirsh O. Seeler J. S. Pichler A. Gast A. Müller S. Miska E. (2002). The SUMO E3 ligase RanBP2 promotes modification of the HDAC4 deacetylase. EMBO J. 21 (11), 2682–2691. 10.1093/emboj/21.11.2682 12032081 PMC125385

[B87] Klempner S. J. Fabrizio D. Bane S. Reinhart M. Peoples T. Ali S. M. (2020). Tumor mutational burden as a predictive biomarker for response to immune checkpoint inhibitors: A review of current evidence. Oncologist 25 (1), e147–e159. 10.1634/theoncologist.2019-0244 31578273 PMC6964127

[B88] Ko J. Y. Chuang P. C. Chen M. W. Ke H. C. Wu S. L. Chang Y. H. (2013). MicroRNA-29a ameliorates glucocorticoid-induced suppression of osteoblast differentiation by regulating β-catenin acetylation. Bone 57 (2), 468–475. 10.1016/j.bone.2013.09.019 24096265

[B89] Kuehl W. M. Bergsagel P. L. (2012). Molecular pathogenesis of multiple myeloma and its premalignant precursor. J. Clin. Invest. 122 (10), 3456–3463. 10.1172/JCI61188 23023717 PMC3461901

[B90] Kuznetsoff J. N. Owens D. A. Lopez A. Rodriguez D. A. Chee N. T. Kurtenbach S. (2021). Dual screen for efficacy and toxicity identifies HDAC inhibitor with distinctive activity spectrum for BAP1-mutant uveal melanoma. Mol. Cancer Res. 19 (2), 215–222. 10.1158/1541-7786.MCR-20-0434 33077485 PMC7864865

[B91] Kuznetsov J. N. Aguero T. H. Owens D. A. Kurtenbach S. Field M. G. Durante M. A. (2019). BAP1 regulates epigenetic switch from pluripotency to differentiation in developmental lineages giving rise to BAP1-mutant cancers. Sci. Adv. 5 (9), eaax1738. 10.1126/sciadv.aax1738 31555735 PMC6750916

[B92] Lahm A. Paolini C. Pallaoro M. Nardi M. C. Jones P. Neddermann P. (2007). Unraveling the hidden catalytic activity of vertebrate class IIa histone deacetylases. Proc. Natl. Acad. Sci. U.S.A. 104 (44), 17335–17340. 10.1073/pnas.0706487104 17956988 PMC2077257

[B93] Lee B. S. Kim Y. S. Kim H. J. Kim D. H. Won H. R. Kim Y. S. (2018). HDAC4 degradation by combined TRAIL and valproic acid treatment induces apoptotic cell death of TRAIL-resistant head and neck cancer cells. Sci. Rep. 8 (1), 12520. 10.1038/s41598-018-31039-8 30131570 PMC6104079

[B94] Lee H. A. Song M. J. Seok Y. M. Kang S. H. Kim S. Y. Kim I. (2015). Histone deacetylase 3 and 4 complex stimulates the transcriptional activity of the mineralocorticoid receptor. PLoS One 10 (8), e0136801. 10.1371/journal.pone.0136801 26305553 PMC4549324

[B95] Lee J. H. Park S. M. Kim O. S. Lee C. S. Woo J. H. Park S. J. (2009). Differential SUMOylation of LXRalpha and LXRbeta mediates transrepression of STAT1 inflammatory signaling in IFN-gamma-stimulated brain astrocytes. Mol. Cell 35 (6), 806–817. 10.1016/j.molcel.2009.07.021 19782030

[B96] Lee Y. Song M. J. Park J. H. Shin M. H. Kim M. K. Hwang D. (2022). Histone deacetylase 4 reverses cellular senescence via DDIT4 in dermal fibroblasts. Aging (Albany NY) 14 (11), 4653–4672. 10.18632/aging.204118 35680564 PMC9217707

[B97] Lensch S. Herschl M. H. Ludwig C. H. Sinha J. Hinks M. M. Mukund A. (2022). Dynamic spreading of chromatin-mediated gene silencing and reactivation between neighboring genes in single cells. Elife 11, e75115. 10.7554/eLife.75115 35678392 PMC9183234

[B98] Li J. Liu C. Li Y. Zheng Q. Xu Y. Liu B. (2019). TMCO1-mediated Ca^2+^ leak underlies osteoblast functions via CaMKII signaling. Nat. Commun. 10 (1), 1589. 10.1038/s41467-019-09653-5 30962442 PMC6453895

[B99] Li P. Wei X. Guan Y. Chen Q. Zhao T. Sun C. (2014). MicroRNA-1 regulates chondrocyte phenotype by repressing histone deacetylase 4 during growth plate development. Faseb J. 28 (9), 3930–3941. 10.1096/fj.13-249318 24858276 PMC4139910

[B100] Li Z. Hassan M. Q. Jafferji M. Aqeilan R. I. Garzon R. Croce C. M. (2009). Biological functions of miR-29b contribute to positive regulation of osteoblast differentiation. J. Biol. Chem. 284 (23), 15676–15684. 10.1074/jbc.M809787200 19342382 PMC2708864

[B101] Lin R. Elf S. Shan C. Kang H. B. Ji Q. Zhou L. (2015). 6-Phosphogluconate dehydrogenase links oxidative PPP, lipogenesis and tumour growth by inhibiting LKB1-AMPK signalling. Nat. Cell Biol. 17 (11), 1484–1496. 10.1038/ncb3255 26479318 PMC4628560

[B102] Little G. H. Bai Y. Williams T. Poizat C. (2007). Nuclear calcium/calmodulin-dependent protein kinase IIdelta preferentially transmits signals to histone deacetylase 4 in cardiac cells. J. Biol. Chem. 282 (10), 7219–7231. 10.1074/jbc.M604281200 17179159

[B227] Liu F. Dowling M. Yang X.-J. Kao G. D. (2004). Caspase-mediated specific cleavage of human histone deacetylase 4. J. Biol. Chem. 279 (33), 34537–34549. 10.1074/jbc.M402475200 15205465

[B103] Liu D. Wu D. Zhao L. Yang Y. Ding J. Dong L. (2015). Arsenic trioxide reduces global histone H4 acetylation at lysine 16 through direct binding to histone acetyltransferase hMOF in human cells. PLoS One 10 (10), e0141014. 10.1371/journal.pone.0141014 26473953 PMC4608833

[B104] Liu R. Wang L. Chen G. Katoh H. Chen C. Liu Y. (2009). FOXP3 up-regulates p21 expression by site-specific inhibition of histone deacetylase 2/histone deacetylase 4 association to the locus. Cancer Res. 69 (6), 2252–2259. 10.1158/0008-5472.CAN-08-3717 19276356 PMC2715174

[B105] Liu Y. Schneider M. F. (2013). Opposing HDAC4 nuclear fluxes due to phosphorylation by β-adrenergic activated protein kinase A or by activity or Epac activated CaMKII in skeletal muscle fibres. J. Physiol. 591 (14), 3605–3623. 10.1113/jphysiol.2013.256263 23652597 PMC3731617

[B106] Lu J. McKinsey T. A. Zhang C. L. Olson E. N. (2000). Regulation of skeletal myogenesis by association of the MEF2 transcription factor with class II histone deacetylases. Mol. Cell 6 (2), 233–244. 10.1016/s1097-2765(00)00025-3 10983972

[B107] Lu Y. Stuart J. H. Talbot-Cooper C. Agrawal-Singh S. Huntly B. Smid A. I. (2019). Histone deacetylase 4 promotes type I interferon signaling, restricts DNA viruses, and is degraded via vaccinia virus protein C6. Proc. Natl. Acad. Sci. U. S. A. 116 (24), 11997–12006. 10.1073/pnas.1816399116 31127039 PMC6575207

[B108] Luan B. Goodarzi M. O. Phillips N. G. Guo X. Chen Y. D. Yao J. (2014). Leptin-mediated increases in catecholamine signaling reduce adipose tissue inflammation via activation of macrophage HDAC4. Cell Metab. 19 (6), 1058–1065. 10.1016/j.cmet.2014.03.024 24768298 PMC4207085

[B109] Macabuag N. Esmieu W. Breccia P. Jarvis R. Blackaby W. Lazari O. (2022). Developing HDAC4-selective protein degraders to investigate the role of HDAC4 in huntington's disease pathology. J. Med. Chem. 65 (18), 12445–12459. 10.1021/acs.jmedchem.2c01149 36098485 PMC9512014

[B110] Marampon F. Megiorni F. Camero S. Crescioli C. McDowell H. P. Sferra R. (2017). HDAC4 and HDAC6 sustain DNA double strand break repair and stem-like phenotype by promoting radioresistance in glioblastoma cells. Cancer Lett. 397, 1–11. 10.1016/j.canlet.2017.03.028 28342984

[B111] Marroncelli N. Bianchi M. Bertin M. Consalvi S. Saccone V. De Bardi M. (2018). HDAC4 regulates satellite cell proliferation and differentiation by targeting P21 and Sharp1 genes. Sci. Rep. 8 (1), 3448. 10.1038/s41598-018-21835-7 29472596 PMC5823886

[B112] Martínez-Zamudio R. I. Roux P. F. de Freitas J. A. N. L. F. Robinson L. Doré G. Sun B. (2020). AP-1 imprints a reversible transcriptional programme of senescent cells. Nat. Cell Biol. 22 (7), 842–855. 10.1038/s41556-020-0529-5 32514071 PMC7899185

[B113] Mathias R. A. Guise A. J. Cristea I. M. (2015). Post-translational modifications regulate class IIa histone deacetylase (HDAC) function in health and disease. Mol. Cell Proteomics 14 (3), 456–470. 10.1074/mcp.O114.046565 25616866 PMC4349969

[B114] McKinsey T. A. Zhang C. L. Olson E. N. (2001). Identification of a signal-responsive nuclear export sequence in class II histone deacetylases. Mol. Cell Biol. 21 (18), 6312–6321. 10.1128/MCB.21.18.6312-6321.2001 11509672 PMC87361

[B115] Menezes M. E. Bhatia S. Bhoopathi P. Das S. K. Emdad L. Dasgupta S. (2014). MDA-7/IL-24: Multifunctional cancer killing cytokine. Adv. Exp. Med. Biol. 818, 127–153. 10.1007/978-1-4471-6458-6_6 25001534 PMC4633013

[B116] Mielcarek M. Zielonka D. Carnemolla A. Marcinkowski J. T. Guidez F. (2015). HDAC4 as a potential therapeutic target in neurodegenerative diseases: A summary of recent achievements. Front. Cell Neurosci. 9, 42. 10.3389/fncel.2015.00042 25759639 PMC4338808

[B117] Mihaylova M. M. Vasquez D. S. Ravnskjaer K. Denechaud P. D. Yu R. T. Alvarez J. G. (2011). Class IIa histone deacetylases are hormone-activated regulators of FOXO and mammalian glucose homeostasis. Cell 145 (4), 607–621. 10.1016/j.cell.2011.03.043 21565617 PMC3117637

[B118] Minisini M. Di Giorgio E. Kerschbamer E. Dalla E. Faggiani M. Franforte E. (2022). Transcriptomic and genomic studies classify NKL54 as a histone deacetylase inhibitor with indirect influence on MEF2-dependent transcription. Nucleic Acids Res. 50 (5), 2566–2586. 10.1093/nar/gkac081 35150567 PMC8934631

[B119] Miska E. A. Karlsson C. Langley E. Nielsen S. J. Pines J. Kouzarides T. (1999). HDAC4 deacetylase associates with and represses the MEF2 transcription factor. EMBO J. 18 (18), 5099–5107. 10.1093/emboj/18.18.5099 10487761 PMC1171580

[B120] Mottet D. Pirotte S. Lamour V. Hagedorn M. Javerzat S. Bikfalvi A. (2009). HDAC4 represses p21(WAF1/Cip1) expression in human cancer cells through a Sp1-dependent, p53-independent mechanism. Oncogene 28 (2), 243–256. 10.1038/onc.2008.371 18850004

[B121] Nakatani T. Chen T. Partridge N. C. (2016). MMP-13 is one of the critical mediators of the effect of HDAC4 deletion on the skeleton. Bone. Sep. 90, 142–151. 10.1016/j.bone.2016.06.010 PMC4970950 27320207

[B122] Nieto M. A. Huang R. Y. Jackson R. A. Thiery J. P. (2016). EMT: 2016. Cell 166 (1), 21–45. 10.1016/j.cell.2016.06.028 27368099

[B123] Nishida N. Mimori K. Fabbri M. Yokobori T. Sudo T. Tanaka F. (2011). MicroRNA-125a-5p is an independent prognostic factor in gastric cancer and inhibits the proliferation of human gastric cancer cells in combination with trastuzumab. Clin. Cancer Res. 17 (9), 2725–2733. 10.1158/1078-0432.CCR-10-2132 21220473

[B124] Niu Y. Wang T. Liu S. Yuan H. Li H. Fu L. (2017). Exercise-induced GLUT4 transcription via inactivation of HDAC4/5 in mouse skeletal muscle in an AMPKα2-dependent manner. Biochim. Biophys. Acta Mol. Basis Dis. 1863 (9), 2372–2381. 10.1016/j.bbadis.2017.07.001 28688716

[B125] Obri A. Makinistoglu M. P. Zhang H. Karsenty G. (2014). HDAC4 integrates PTH and sympathetic signaling in osteoblasts. J. Cell Biol. 205 (6), 771–780. 10.1083/jcb.201403138 24934156 PMC4068141

[B126] Osanai T. Tanaka M. Mikami K. Kitajima M. Tomisawa T. Magota K. (2018). Novel anti-aging gene NM_026333 contributes to proton-induced aging via NCX1-pathway. J. Mol. Cell Cardiol. 125, 174–184. 10.1016/j.yjmcc.2018.10.021 30385152

[B127] Ozcan L. Ghorpade D. S. Zheng Z. de Souza J. C. Chen K. Bessler M. (2016). Hepatocyte DACH1 is increased in obesity via nuclear exclusion of HDAC4 and promotes hepatic insulin resistance. Cell Rep. 15 (10), 2214–2225. 10.1016/j.celrep.2016.05.006 27239042 PMC5068925

[B128] Pablo Tortola C. Fielitz B. Li Y. Rüdebusch J. Luft F. C. Fielitz J. (2021). Activation of tripartite motif containing 63 expression by transcription factor EB and transcription factor binding to immunoglobulin heavy chain enhancer 3 is regulated by protein kinase D and class IIa histone deacetylases. Front. Physiol. 11, 550506. 10.3389/fphys.2020.550506 33519497 PMC7838639

[B129] Paluvai H. Di Giorgio E. Brancolini C. (2018). Unscheduled HDAC4 repressive activity in human fibroblasts triggers TP53-dependent senescence and favors cell transformation. Mol. Oncol. 12 (12), 2165–2181. 10.1002/1878-0261.12392 30315623 PMC6275271

[B130] Pan L. Pan H. Jiang H. Du J. Wang X. Huang B. (2010). HDAC4 inhibits the transcriptional activation of mda-7/IL-24 induced by Sp1. Cell Mol. Immunol. 7 (3), 221–226. 10.1038/cmi.2010.12 20383178 PMC4002910

[B131] Park S. Y. Kim G. S. Hwang H. J. Nam T. H. Park H. S. Song J. (2018). Structural basis of the specific interaction of SMRT corepressor with histone deacetylase 4. Nucleic Acids Res. 46 (22), 11776–11788. 10.1093/nar/gky926 30321390 PMC6294515

[B228] Paroni G. Mizzau M. Henderson C. Del Sal G. Schneider C. Brancolini C. (2018). Caspase-dependent regulation of histone deacetylase 4 nuclear-cytoplasmic shuttling promotes apoptosis. Mol. Biol. Cell 15 (6), 2804–2818. 10.1091/mbc.e03-08-0624 PMC420104 15075374

[B132] Paroni G. Cernotta N. Dello Russo C. Gallinari P. Pallaoro M. Foti C. (2008). PP2A regulates HDAC4 nuclear import. Mol. Biol. Cell. Feb 19 (2), 655–667. 10.1091/mbc.e07-06-0623 PMC2230598 18045992

[B133] Paroni G. Fontanini A. Cernotta N. Foti C. Gupta M. P. Yang X. J. (2007). Dephosphorylation and caspase processing generate distinct nuclear pools of histone deacetylase 4. Mol. Cell Biol. 27 (19), 6718–6732. 10.1128/MCB.00853-07 17636017 PMC2099224

[B134] Parra M. Verdin E. (2010). Regulatory signal transduction pathways for class IIa histone deacetylases. Curr. Opin. Pharmacol. 10 (4), 454–460. 10.1016/j.coph.2010.04.004 20447866

[B135] Pei X. F. Cao L. L. Huang F. Qiao X. Yu J. Ye H. (2018). Role of miR-22 in intestinal mucosa tissues and peripheral blood CD4+ T cells of inflammatory bowel disease. Pathol. Res. Pract. 214 (8), 1095–1104. 10.1016/j.prp.2018.04.009 29880327

[B136] Peng T. Ji D. Jiang Y. (2021). Long non-coding RNA GAS5 suppresses rheumatoid arthritis progression via miR-128-3p/HDAC4 axis. Mol. Cell Biochem. 476 (6), 2491–2501. 10.1007/s11010-021-04098-1 33611674

[B137] Peruzzo P. Comelli M. Di Giorgio. E. Franforte E. Mavelli I. Brancolini C. (2016). Transformation by different oncogenes relies on specific metabolic adaptations. Cell Cycle 15 (19), 2656–2668. 10.1080/15384101.2016.1215387 27485932 PMC5053564

[B138] Pigna E. Renzini A. Greco E. Simonazzi E. Fulle S. Mancinelli R. (2018). HDAC4 preserves skeletal muscle structure following long-term denervation by mediating distinct cellular responses. Skelet. Muscle 8 (1), 6. 10.1186/s13395-018-0153-2 29477142 PMC6389241

[B139] Potthoff M. J. Wu H. Arnold M. A. Shelton J. M. Backs J. McAnally J. (2007). Histone deacetylase degradation and MEF2 activation promote the formation of slow-twitch myofibers. J. Clin. Invest. 117 (9), 2459–2467. 10.1172/JCI31960 17786239 PMC1957540

[B140] Qian D. Z. Kachhap S. K. Collis S. J. Verheul H. M. Carducci M. A. Atadja P. (2006). Class II histone deacetylases are associated with VHL-independent regulation of hypoxia-inducible factor 1 alpha. Cancer Res. 66 (17), 8814–8821. 10.1158/0008-5472.CAN-05-4598 16951198

[B141] Rad R. Rad L. Wang W. Cadinanos J. Vassiliou G. Rice S. (2010). PiggyBac transposon mutagenesis: A tool for cancer gene discovery in mice. Science 330 (6007), 1104–1107. 10.1126/science.1193004 20947725 PMC3719098

[B142] Ren J. Huang H. J. Gong Y. Yue S. Tang L. M. Cheng S. Y. (2014). MicroRNA-206 suppresses gastric cancer cell growth and metastasis. Cell Biosci. 4, 26. 10.1186/2045-3701-4-26 24855559 PMC4030529

[B143] Renzini A. Marroncelli N. Cavioli G. Di Francescantonio S. Forcina L. Lambridis A. (2022). Cytoplasmic HDAC4 regulates the membrane repair mechanism in Duchenne muscular dystrophy. J. Cachexia Sarcopenia Muscle 13 (2), 1339–1359. 10.1002/jcsm.12891 35170869 PMC8977968

[B144] Roccaro A. M. Sacco A. Jia X. Azab A. K. Maiso P. Ngo H. T. (2010). microRNA-dependent modulation of histone acetylation in Waldenstrom macroglobulinemia. Blood 116 (9), 1506–1514. 10.1182/blood-2010-01-265686 20519629 PMC2938840

[B145] Rosenbloom K. R. Dreszer T. R. Pheasant M. Barber G. P. Meyer L. R. Pohl A. (2009). ENCODE whole-genome data in the UCSC Genome Browser. Nucleic Acids Res. 38, D620–D625. 10.1093/nar/gkp961 19920125 PMC2808953

[B146] Sakamoto K. Bultot L. Göransson O. (2018). The salt-inducible kinases: Emerging metabolic regulators. Trends Endocrinol. Metab. 29 (12), 827–840. 10.1016/j.tem.2018.09.007 30385008

[B147] Salmena L. Poliseno L. Tay Y. Kats L. Pandolfi P. P. (2011). A ceRNA hypothesis: The rosetta stone of a hidden RNA language? Cell 146 (3), 353–358. 10.1016/j.cell.2011.07.014 21802130 PMC3235919

[B148] Salminen A. Kauppinen A. Kaarniranta K. (2016). AMPK/Snf1 signaling regulates histone acetylation: Impact on gene expression and epigenetic functions. Cell Signal 28 (8), 887–895. 10.1016/j.cellsig.2016.03.009 27010499

[B149] Sandhu S. K. Volinia S. Costinean S. Galasso M. Neinast R. Santhanam R. (2012). miR-155 targets histone deacetylase 4 (HDAC4) and impairs transcriptional activity of B-cell lymphoma 6 (BCL6) in the Eμ-miR-155 transgenic mouse model. Proc. Natl. Acad. Sci. U. S. A. 109 (49), 20047–20052. d oi:. 10.1073/pnas.1213764109 23169640 PMC3523868

[B150] Sato T. Verma S. Andrade C. D. C. Omeara M. Campbell N. Wang J. S. (2020). A FAK/HDAC5 signaling axis controls osteocyte mechanotransduction. Nat. Commun. 11 (1), 3282. 10.1038/s41467-020-17099-3 32612176 PMC7329900

[B151] Saxton R. A. Sabatini D. M. (2017). mTOR signaling in growth, metabolism, and disease. Cell 168 (6), 960–976. 10.1016/j.cell.2017.02.004 28283069 PMC5394987

[B152] Schader T. Löwe O. Reschke C. Malacarne P. Hahner F. Müller N. (2020). Oxidation of HDAC4 by Nox4-derived H2O2 maintains tube formation by endothelial cells. Redox Biol. 36, 101669. 10.1016/j.redox.2020.101669 32818796 PMC7452117

[B153] Semenza G. L. (2012). Hypoxia-inducible factors in physiology and medicine. Cell 148 (3), 399–408. 10.1016/j.cell.2012.01.021 22304911 PMC3437543

[B154] Semenza G. L. (2016). Hypoxia-inducible factors: Coupling glucose metabolism and redox regulation with induction of the breast cancer stem cell phenotype. EMBO J. 36 (3), 252–259. 10.15252/embj.201695204 28007895 PMC5286373

[B155] Seo H. W. Kim E. J. Na H. Lee M. O. (2009). Transcriptional activation of hypoxia-inducible factor-1alpha by HDAC4 and HDAC5 involves differential recruitment of p300 and FIH-1. FEBS Lett. 583 (1), 55–60. 10.1016/j.febslet.2008.11.044 19071119

[B156] Shah S. P. Lonial S. Boise L. H. (2015). When cancer fights back: Multiple myeloma, proteasome inhibition, and the heat-shock response. Mol. Cancer Res. 13 (8), 1163–1173. 10.1158/1541-7786.MCR-15-0135 26013169 PMC4874259

[B157] Shan C. Elf S. Ji Q. Kang H. B. Zhou L. Hitosugi T. (2014). Lysine acetylation activates 6-phosphogluconate dehydrogenase to promote tumor growth. Mol. Cell 55 (4), 552–565. 10.1016/j.molcel.2014.06.020 25042803 PMC4142084

[B158] Shimizu E. Nakatani T. He Z. Partridge N. C. (2014). Parathyroid hormone regulates histone deacetylase (HDAC) 4 through protein kinase A-mediated phosphorylation and dephosphorylation in osteoblastic cells. J. Biol. Chem. 289 (31), 21340–21350. 10.1074/jbc.M114.550699 24904057 PMC4118099

[B159] Singh A. Happel C. Manna S. K. Acquaah-Mensah G. Carrerero J. Kumar S. (2013). Transcription factor NRF2 regulates miR-1 and miR-206 to drive tumorigenesis. J. Clin. Invest. 123 (7), 2921–2934. 10.1172/JCI66353 23921124 PMC3696551

[B160] Singh R. Letai A. Sarosiek K. (2019). Regulation of apoptosis in health and disease: The balancing act of BCL-2 family proteins. Nat. Rev. Mol. Cell Biol. 20 (3), 175–193. 10.1038/s41580-018-0089-8 30655609 PMC7325303

[B161] Sinnett-Smith J. Ni Y. Wang J. Ming M. Young S. H. Rozengurt E. (2014). Protein kinase D1 mediates class IIa histone deacetylase phosphorylation and nuclear extrusion in intestinal epithelial cells: Role in mitogenic signaling. Am. J. Physiol. Cell Physiol. 306 (10), C961–C971. 10.1152/ajpcell.00048.2014 24647541 PMC4024715

[B162] Song B. Wang Y. Xi Y. Kudo K. Bruheim S. Botchkina G. I. (2009). Mechanism of chemoresistance mediated by miR-140 in human osteosarcoma and colon cancer cells. Oncogene 28 (46), 4065–4074. 10.1038/onc.2009.274 19734943 PMC2783211

[B163] Sparrow D. B. Miska E. A. Langley E. Reynaud-Deonauth S. Kotecha S. Towers N. (1999). MEF-2 function is modified by a novel co-repressor, MITR. EMBO J. 18 (18), 5085–5098. 10.1093/emboj/18.18.5085 10487760 PMC1171579

[B164] Su L. Luo Y. Yang Z. Yang J. Yao C. Cheng F. (2016). MEF2D transduces microenvironment stimuli to ZEB1 to promote epithelial-mesenchymal transition and metastasis in colorectal cancer. Cancer Res. 76 (17), 5054–5067. 10.1158/0008-5472.CAN-16-0246 27364559

[B165] Sun Y. Ge Y. Drnevich J. Zhao Y. Band M. Chen J. (2010). Mammalian target of rapamycin regulates miRNA-1 and follistatin in skeletal myogenesis. J. Cell Biol. 189 (7), 1157–1169. 10.1083/jcb.200912093 20566686 PMC2894448

[B224] Szklarczyk D. Gable A. L. Nastou K. C. Lyon D. Kirsch R. Pyysalo S. (2021). The STRING database in 2021: Customizable protein-protein networks, and functional characterization of user-uploaded gene/measurement sets. Nucleic Acids Res. 49 (D1), D605–D612. 10.1093/nar/gkaa1074 33237311 PMC7779004

[B166] Tan X. Banerjee P. Liu X. Yu J. Lee S. Ahn Y. H. (2022). Transcriptional control of a collagen deposition and adhesion process that promotes lung adenocarcinoma growth and metastasis. JCI Insight 7 (1), e153948. 10.1172/jci.insight.153948 34874914 PMC8765047

[B167] Tarumoto Y. Lin S. Wang J. Milazzo J. P. Xu Y. Lu B. (2020). Salt-inducible kinase inhibition suppresses acute myeloid leukemia progression *in vivo* . Blood 135 (1), 56–70. 10.1182/blood.2019001576 31697837 PMC6940199

[B168] Tarumoto Y. Lu B. Somerville T. D. D. Huang Y. H. Milazzo J. P. Wu X. S. (2018). LKB1, salt-inducible kinases, and MEF2C are linked dependencies in acute myeloid leukemia. Mol. Cell 69 (6), 1017–1027. e6. 10.1016/j.molcel.2018.02.011 29526696 PMC5856641

[B169] Taylor M. V. Hughes S. M. (2017). Mef2 and the skeletal muscle differentiation program. Semin. Cell Dev. Biol. 72, 33–44. 10.1016/j.semcdb.2017.11.020 29154822

[B170] Torchy M. P. Hamiche A. Klaholz B. P. (2015). Structure and function insights into the NuRD chromatin remodeling complex. Cell Mol. Life Sci. 72 (13), 2491–2507. 10.1007/s00018-015-1880-8 25796366 PMC11114056

[B171] Tsai C. L. Liu W. L. Hsu F. M. Yang P. S. Yen R. F. Tzen K. Y. (2018). Targeting histone deacetylase 4/ubiquitin-conjugating enzyme 9 impairs DNA repair for radiosensitization of hepatocellular carcinoma cells in mice. Hepatology 67 (2), 586–599. 10.1002/hep.29328 28646552

[B172] Vallabhapurapu S. D. Noothi S. K. Pullum D. A. Lawrie C. H. Pallapati R. Potluri V. (2015). Transcriptional repression by the HDAC4-RelB-p52 complex regulates multiple myeloma survival and growth. Nat. Commun. 6, 8428. 10.1038/ncomms9428 26455434

[B173] Valsecchi V. Boido M. De Amicis E. Piras A. Vercelli A. (2015). E xpression of muscle-specific MiRNA 206 in the progression of disease in a murine SMA model. PLoS One 0 (6), e0128560. 10.1371/journal.pone.0128560 PMC4450876 26030275

[B174] Varadi M. Anyango S. Deshpande M. Nair S. Natassia C. Yordanova G. (2022). AlphaFold protein structure database: Massively expanding the structural coverage of protein-sequence space with high-accuracy models. Nucleic Acids Res. 50 (D1), D439–D444. 10.1093/nar/gkab1061 34791371 PMC8728224

[B175] Vega R. B. Matsuda K. Oh J. Barbosa A. C. Yang X. Meadows E. (2004). Histone deacetylase 4 controls chondrocyte hypertrophy during skeletogenesis. Cell 119 (4), 555–566. 10.1016/j.cell.2004.10.024 15537544

[B176] Velasco-Aviles S. Patel N. Casillas-Bajo A. Frutos-Rincón L. Velasco E. Gallar J. (2022). A genetic compensatory mechanism regulated by *Jun* and *Mef2d* modulates the expression of distinct class IIa *Hdacs* to ensure peripheral nerve myelination and repair. Elife 11, e72917. 10.7554/eLife.72917 35076395 PMC8853665

[B177] Veloso A. Martin M. Bruyr J. O'Grady T. Deroanne C. Mottet D. (2019). Dephosphorylation of HDAC4 by PP2A-Bδ unravels a new role for the HDAC4/MEF2 axis in myoblast fusion. Cell Death Dis. 10 (7), 512. 10.1038/s41419-019-1743-6 31273193 PMC6609635

[B178] Villavicencio-Lorini P. Klopocki E. Trimborn M. Koll R. Mundlos S. Horn D. (2013). Phenotypic variant of Brachydactyly-mental retardation syndrome in a family with an inherited interstitial 2q37.3 microdeletion including HDAC4. Eur. J. Hum. Genet. 21 (7), 743–748. 10.1038/ejhg.2012.240 23188045 PMC3722943

[B179] Wakeling E. McEntagart M. Bruccoleri M. Shaw-Smith C. Stals K. L. Wakeling M. (2021). Missense substitutions at a conserved 14-3-3 binding site in HDAC4 cause a novel intellectual disability syndrome. HGG Adv. 14 (21), 100015. 10.1016/j.xhgg.2020.100015 PMC7841527 33537682

[B180] Walkinshaw D. R. Weist R. Kim G. W. You L. Xiao L. Nie J. (2013). The tumor suppressor kinase LKB1 activates the downstream kinases SIK2 and SIK3 to stimulate nuclear export of class IIa histone deacetylases. J. Biol. Chem. 288 (13), 9345–9362. 10.1074/jbc.M113.456996 23393134 PMC3611005

[B181] Wang A. H. Kruhlak M. J. Wu J. Bertos N. R. Vezmar M. Posner B. I. (2000). Regulation of histone deacetylase 4 by binding of 14-3-3 proteins. Mol. Cell Biol. 20 (18), 6904–6912. 10.1128/MCB.20.18.6904-6912.2000 10958686 PMC88766

[B182] Wang A. H. Yang X. J. (2001). Histone deacetylase 4 possesses intrinsic nuclear import and export signals. Mol. Cell Biol. 21 (17), 5992–6005. 10.1128/MCB.21.17.5992-6005.2001 11486037 PMC87317

[B183] Wang B. Li D. Filkowski J. Rodriguez-Juarez R. Storozynsky Q. Malach M. (2018). A dual role of miR-22 modulated by RelA/p65 in resensitizing fulvestrant-resistant breast cancer cells to fulvestrant by targeting FOXP1 and HDAC4 and constitutive acetylation of p53 at Lys382. Oncogenesis 7 (7), 54. 10.1038/s41389-018-0063-5 30057418 PMC6064715

[B184] Wang B. Liu T. Y. Lai C. H. Rao Y. H. Choi M. C. Chi J. T. (2014). Glycolysis-dependent histone deacetylase 4 degradation regulates inflammatory cytokine production. Mol. Biol. Cell 25 (21), 3300–3307. 10.1091/mbc.E13-12-0757 25187650 PMC4214777

[B185] Wang B. Moya N. Niessen S. Hoover H. Mihaylova M. M. Shaw R. J. (2011). A hormone-dependent module regulating energy balance. Cell 145 (4), 596–606. 10.1016/j.cell.2011.04.013 21565616 PMC3129781

[B186] Wang F. Marshall C. B. Ikura M. (2013). Transcriptional/epigenetic regulator CBP/p300 in tumorigenesis: Structural and functional versatility in target recognition. Cell Mol. Life Sci. 70 (21), 3989–4008. 10.1007/s00018-012-1254-4 23307074 PMC11113169

[B187] Wang W. Pan K. Chen Y. Huang C. Zhang X. (2012). The acetylation of transcription factor HBP1 by p300/CBP enhances p16INK4A expression. Nucleic Acids Res. 40 (3), 981–995. 10.1093/nar/gkr818 21967847 PMC3273810

[B188] WangZ. Qin G. Zhao T. C. (2014). HDAC4: Mechanism of regulation and biological functions. Epigenomics 6 (1), 139–150. 10.2217/epi.13.73 24579951 PMC4380265

[B189] Wei X. Li H. Zhang B. Li C. Dong D. Lan X. (2016). miR-378a-3p promotes differentiation and inhibits proliferation of myoblasts by targeting HDAC4 in skeletal muscle development. RNA Biol. 13 (12), 1300–1309. 10.1080/15476286.2016.1239008 27661135 PMC5207390

[B190] Wheeler P. G. Huang D. Dai Z. (2014). Haploinsufficiency of HDAC4 does not cause intellectual disability in all affected individuals. Am. J. Med. Genet. A 164A (7), 1826–1829. 10.1002/ajmg.a.36542 24715439

[B191] White E. DiPaola R. S. (2009). The double-edged sword of autophagy modulation in cancer. Clin. Cancer Res. 15 (17), 5308–5316. 10.1158/1078-0432.CCR-07-5023 19706824 PMC2737083

[B192] Williams A. H. Valdez G. Moresi V. Qi X. McAnally J. Elliot J. L. (2009). MicroRNA-206 delays ALS progression and promotes regeneration of neuromuscular synapses in mice. Science 326 (5959), 1549–1554. 10.1126/science.1181046 20007902 PMC2796560

[B193] Williams S. R. Aldred M. A. Der Kaloustian V. M. Halal F. Gowans G. McLeod D. R. (2010). Haploinsufficiency of HDAC4 causes brachydactyly mental retardation syndrome, with brachydactyly type E, developmental delays, and behavioral problems. Am. J. Hum. Genet. 87 (2), 219–228. 10.1016/j.ajhg.2010.07.011 20691407 PMC2917703

[B194] Wilson A. J. Byun D. S. Nasser S. Murray L. B. Ayyanar K. Arango D. (2008). HDAC4 promotes growth of colon cancer cells via repression of p21. Mol. Biol. Cell 19 (10), 4062–4075. 10.1091/mbc.e08-02-0139 18632985 PMC2555950

[B195] Winbanks C. E. Wang B. Beyer C. Koh P. White L. Kantharidis P. (2011). TGF-beta regulates miR-206 and miR-29 to control myogenic differentiation through regulation of HDAC4. J. Biol. Chem. 286 (16), 13805–13814. 10.1074/jbc.M110.192625 21324893 PMC3077581

[B196] Wu H. Liu C. Yang Q. Xin C. Du J. Sun F. (2020). MIR145-3p promotes autophagy and enhances bortezomib sensitivity in multiple myeloma by targeting HDAC4. Autophagy 16 (4), 683–697. 10.1080/15548627.2019.1635380 31242129 PMC7138223

[B197] Wu Y. Hou F. Wang X. Kong Q. Han X. Bai B. (2016). Aberrant expression of histone deacetylases 4 in cognitive disorders: Molecular mechanisms and a potential target. Front. Mol. Neurosci. 9, 114. 10.3389/fnmol.2016.00114 27847464 PMC5088184

[B198] Xiao L. Gong D. Liang L. Liang A. Liang H. Xu X. (2021). Inhibition of HDAC4 by GSK3β leads to downregulation of KLF5 and ASK1 and prevents the progression of intravertebral disc degeneration. Clin. Epigenetics 13 (1), 53. 10.1186/s13148-021-01005-9 33691773 PMC7948391

[B199] Xiao Q. Gan Y. Li Y. Fan L. Liu J. Lu P. (2021). MEF2A transcriptionally upregulates the expression of ZEB2 and CTNNB1 in colorectal cancer to promote tumor progression. Oncogene 40 (19), 3364–3377. 10.1038/s41388-021-01774-w 33863999 PMC8116210

[B200] Xing T. Zhu J. Xian J. Li A. Wang X. Wang W. (2019). miRNA-548ah promotes the replication and expression of Hepatitis B virus by targeting histone deacetylase 4. Life Sci. 219, 199–208. 10.1016/j.lfs.2018.12.057 30615846

[B201] Xu D. Gao Y. Hu N. Wu L. Chen Q. (2017). miR-365 ameliorates dexamethasone-induced suppression of osteogenesis in mc3t3-E1 cells by targeting HDAC4. Int. J. Mol. Sci. 18 (5), 977. 10.3390/ijms18050977 28471397 PMC5454890

[B202] Yang D. Xiao C. Long F. Su Z. Jia W. Qin M. (2018). HDAC4 regulates vascular inflammation via activation of autophagy. Cardiovasc Res. 114 (7), 1016–1028. 10.1093/cvr/cvy051 29529137

[B203] Yang Q. Tang J. Xu C. Zhao H. Zhou Y. Wang Y. (2020). Histone deacetylase 4 inhibits NF-κB activation by facilitating IκBα sumoylation. J. Mol. Cell Biol. 12 (12), 933–945. 10.1093/jmcb/mjaa043 32770227 PMC7948076

[B204] Yim J. H. Baek J. H. Lee C. W. Kim M. J. Yun H. S. Hong E. H. (2013). Identification of HDAC4 as a target of γ-catenin that regulates the oncogenic K-Ras-mediated malignant phenotype of Rat2 cells. Biochem. Biophys. Res. Commun. 436 (3), 436–442. 10.1016/j.bbrc.2013.05.122 23747726

[B205] Yu Y. Dong G. Li Z. Zheng Y. Shi Z. Wang G. (2022). circ-LRP6 contributes to osteosarcoma progression by regulating the miR-141-3p/HDAC4/HMGB1 axis. Int. J. Oncol. 60 (4), 38. 10.3892/ijo.2022.5328 35211755 PMC8878724

[B206] Yuan H. Denton K. Liu L. Li X. J. Benashski S. McCullough L. (2016). Nuclear translocation of histone deacetylase 4 induces neuronal death in stroke. Neurobiol. Dis. 91, 182–193. 10.1016/j.nbd.2016.03.004 26969532

[B207] Yuan J. Yang F. Chen B. Lu Z. Huo X. Zhou W. (2011). The histone deacetylase 4/SP1/microrna-200a regulatory network contributes to aberrant histone acetylation in hepatocellular carcinoma. Hepatology 54 (6), 2025–2035. 10.1002/hep.24606 21837748

[B208] Yue F. Li W. Zou J. Chen Q. Xu G. Huang H. (2015). Blocking the association of HDAC4 with MAP1S accelerates autophagy clearance of mutant Huntingtin. Aging (Albany NY) 7 (10), 839–853. 10.18632/aging.100818 26540094 PMC4637209

[B209] Zang W. J. Hu Y. L. Qian C. Y. Feng Y. Liu J. Z. Yang J. L. (2022). HDAC4 promotes the growth and metastasis of gastric cancer via autophagic degradation of MEKK3. Br. J. Cancer 127 (2), 237–248. 10.1038/s41416-022-01805-7 35637410 PMC9296555

[B210] Zeng L. S. Yang X. Z. Wen Y. F. Mail S. J. Wang M. H. Zhang M. Y. (2016). Overexpressed HDAC4 is associated with poor survival and promotes tumor progression in esophageal carcinoma. Aging (Albany NY) 8 (6), 1236–1249. 10.18632/aging.100980 27295551 PMC4931829

[B211] Zeng Y. Yin L. Zhou J. Zeng R. Xiao Y. Black A. R. (2022). MARK2 regulates chemotherapeutic responses through class IIa HDAC-YAP axis in pancreatic cancer. Oncogene 41 (31), 3859–3875. 10.1038/s41388-022-02399-3 35780183 PMC9339507

[B212] Zhang J. Yang Y. Yang T. Liu Y. Li A. Fu S. (2010). microRNA-22, downregulated in hepatocellular carcinoma and correlated with prognosis, suppresses cell proliferation and tumourigenicity. Br. J. Cancer 103 (8), 1215–1220. 10.1038/sj.bjc.6605895 20842113 PMC2967065

[B213] Zhang L. Sun Y. Fei M. Tan C. Wu J. Zheng J. (2014). Disruption of chaperone-mediated autophagy-dependent degradation of MEF2A by oxidative stress-induced lysosome destabilization. Autophagy 10 (6), 1015–1035. 10.4161/auto.28477 24879151 PMC4091166

[B214] Zhang M. Wei Y. Liu Y. Guan W. Zhang X. Kong J. (2020). Metastatic phosphatase PRL-3 induces Ovaria.n cancer stem cell sub-population through phosphatase-independent deacetylation modulations. IScience 23 (1), 100766. 10.1016/j.isci.2019.100766 31887658 PMC6941878

[B215] Zhang Y. Ren Y. J. Guo L. C. Ji C. Hu J. Zhang H. H. (2017). Nucleus accumbens-associated protein-1 promotes glycolysis and survival of hypoxic tumor cells via the HDAC4-HIF-1α axis. Oncogene 36 (29), 4171–4181. 10.1038/onc.2017.51 28319066 PMC5537617

[B216] Zhao L. Chen H. Zhang Q. Y. Ma J. Hu H. Xu L. (2022). ATF4-mediated microRNA-145/HDAC4/p53 axis affects resistance of colorectal cancer cells to 5-fluorouracil by regulating autophagy. Cancer Chemother. Pharmacol. 89 (5), 595–607. 10.1007/s00280-021-04393-0 35312836

[B217] Zhao X. Ito A. Kane C. D. Liao T. S. Bolger T. A. Lemrow S. M. (2001). The modular nature of histone deacetylase HDAC4 confers phosphorylation-dependent intracellular trafficking. J. Biol. Chem. 276 (37), 35042–35048. 10.1074/jbc.M105086200 11470791

[B218] Zhao X. Shen J. Zhao X. Zhang M. Feng X. Zhang W. (2022). PIM3-AMPK-HDAC4/5 axis restricts MuERVL-marked 2-cell-like state in embryonic stem cells. Stem Cell Rep. 17 (10), 2256–2271. 10.1016/j.stemcr.2022.08.009 PMC9561635 36150380

[B219] Zhao X. Sternsdorf T. Bolger T. A. Evans R. M. Yao T. P. (2005). Regulation of MEF2 by histone deacetylase 4- and SIRT1 deacetylase-mediated lysine modifications. Mol. Cell Biol. 25 (19), 8456–8464. 10.1128/MCB.25.19.8456-8464.2005 16166628 PMC1265742

[B220] Zhou J. Li P. Chen Q. Wei X. Zhao T. Wang Z. (2015). Mitogen-activated protein kinase p38 induces HDAC4 degradation in hypertrophic chondrocytes. Biochim. Biophys. Acta 1853 (2), 370–376. 10.1016/j.bbamcr.2014.11.003 25447540 PMC4289442

[B221] Zhou L. Zheng S. Rosas Bringas F. R. Bakker B. Simon J. E. Bakker P. L. (2021). A synthetic lethal screen identifies HDAC4 as a potential target in MELK overexpressing cancers. G3 (Bethesda) 11 (12), jkab335. 10.1093/g3journal/jkab335 34550356 PMC8664443

[B222] Zhu L. Yang J. Zhao L. Yu X. Wang L. Wang F. (2015). Expression of hMOF, but not HDAC4, is responsible for the global histone H4K16 acetylation in gastric carcinoma. Int. J. Oncol. 46 (6), 2535–2545. 10.3892/ijo.2015.2956 25873202

